# A scoping review of interprofessional education in healthcare: evaluating competency development, educational outcomes and challenges

**DOI:** 10.1186/s12909-025-06969-3

**Published:** 2025-03-20

**Authors:** Hemal Patel, Simone Perry, Eric Badu, Felista Mwangi, Oyepeju Onifade, Alexander Mazurskyy, Joanne Walters, Meredith Tavener, Danielle Noble, Sherphard Chidarikire, Lee Lethbridge, Liam Jobson, Hamish Carver, Annabelle MacLellan, Natalie Govind, Graham Andrews, Greg Kerrison-Watkin, Elizabeth Lun, Bunmi S. Malau-Aduli, on behalf of the ACHIEVE Network

**Affiliations:** 1 School of Medicine and Public Health, College of Health, Medicine and Wellbeing, University of Newcastle, Callaghan, NSW 2308 Australia; 2 Central Coast Local Health District, Gosford, NSW 2250 Australia; 3 New South Wales Ambulance, Rozelle, NSW 2039 Australia; 4 Social Policy Research Centre, The University of New South Wales, Sydney, NSW 2052 Australia; 5 College of Medicine and Dentistry, James Cook University, Townsville, QLD 4812 Australia; 6 School of Health Sciences, College of Health, Medicine and Wellbeing, University of Newcastle, Callaghan, NSW 2308 Australia; 7 School of Nursing and Midwifery, College of Health, Medicine and Wellbeing, University of Newcastle, Callaghan, NSW 2308 Australia; 8 School of Nursing and Midwifery, University of Technology Sydney, Ultimo, NSW 2007 Australia; 9 Central Coast Local Health District, Wyong, NSW 2259 Australia; 10 School of Rural Medicine, Faculty of Medicine and Health, University of New England, Armidale, NSW 2350 Australia

**Keywords:** Core competencies, Health professions education, Healthcare students, Interprofessional communication, Interprofessional learning, Roles and responsibilities, Teamwork, Values for interprofessional practice

## Abstract

**Background:**

Interprofessional education (IPE) is essential in healthcare to enhance collaboration, communication and teamwork among health professions education students. This review aimed to map out the core competencies health professions education students develop during IPE and identify the positive and negative educational outcomes.

**Methods:**

A comprehensive search strategy was developed and reported in accordance with the PRISMA ScR guidelines. The search was conducted across five electronic databases (Medline, Scopus, Web of Science, PsycINFO and EBSCO) for peer-reviewed articles published in English within the last 20 years. Data was extracted and core competencies were categorised into four defined areas—roles and responsibilities; interprofessional communication; values for interprofessional practice; teams and teamwork. The frequency of occurrence of each core competency, along with the positive and negative outcomes of IPE were analysed. Mixed methods analysis was used to integrate both qualitative and quantitative data.

**Results:**

Team and teamwork emerged as the most frequently attained core competency in IPE. The positive impacts of IPE include significant improvements in role clarity, communication skills, and teamwork dynamics. However, negative impacts were also noted, such as logistical challenges and interpersonal issues like power dynamics and communication barriers, which impeded the personal professional growth and professional interactional skill-related benefits of IPE. Additionally, some participants reported feeling overwhelmed by the extra workload required for IPE activities.

**Conclusion:**

IPE is a valuable component of health professions education, significantly contributing to the development of core competencies necessary for interprofessional collaborative practice. Addressing the challenges and implementing best practices can further enhance the effectiveness of IPE programs, ultimately improving healthcare outcomes. The implications for practice, training of healthcare students and future research are discussed.

**Supplementary Information:**

The online version contains supplementary material available at 10.1186/s12909-025-06969-3.

## Introduction

Interprofessional learning (IPL) – the “learning that arises from the interaction between members of two or more professions”– [1] is increasingly recognised as a critical component in the education of healthcare students and professionals. IPL may develop through interprofessional education (IPE) or during interactions in educational or clinical practice settings [1]. According to the World Health Organisation (WHO), IPE “occurs when students from two or more professions learn about, from, and with each other” [2] to develop skills that are essential for interprofessional collaborative practice. IPE focuses on collaborative learning, engagement and education among healthcare students to achieve common goals that enhance patient experience and wellbeing [3, 4]. Students from different healthcare professions such as medicine, nursing and allied health work together, aiming to improve patient outcomes through shared educational experiences [2, 4, 5]. Interprofessional Practice (IPP) is the application of interprofessional collaboration in clinical practice settings, where professionals from various professions practice together to provide comprehensive care [2]. IPP involves the application of collaborative skills in clinical practice settings, when health professionals from diverse fields like medicine, nursing and allied health work together to deliver comprehensive services, ensuring the delivery of the highest quality of care to patients, their families, carers, and communities [4]. While the terms IPL and IPE are often used interchangeably, the term IPE will be used for the purpose of this review.

The wellbeing of health professionals often hinges on their ability to work together seamlessly. IPP has emerged as a crucial element in enhancing job satisfaction, reducing stress, and preventing burnout among healthcare workers [6, 7]. Empirical studies have highlighted these benefits, emphasising shared goals and a person-centred approach to care [6–8]. This approach improves access to health interventions, supports coordination and enhances involvement in decision-making [9]. The approach also strengthens health systems, making them more resilient, comprehensive, and responsive to population needs [10]. The significance of these interactions goes beyond individual experiences. When healthcare providers collaborate effectively, they create a supportive and efficient work environment. This cultural shift towards collaboration starts in educational institutions where students from various health professions learn together, fostering mutual respect and understanding. This culture, nurtured through IPE, is essential for transforming healthcare settings into places where teamwork is the norm, not the exception. This makes it imperative for health education institutions to embed IPE within their curricula and healthcare programs.

IPE has been widely studied and recognised for its benefits in healthcare education and practice [11–13]. IPE has been reported as pivotal in preparing students for the health workforce, where teamwork is an essential competency required for practice [12]. Numerous studies have demonstrated that IPE fosters interprofessional collaboration, reduces barriers and preconceptions among different healthcare student groups and enhances professional competencies [13–15]. Nonetheless, the complexity of teaching dynamics in various healthcare professions presents unique challenges to the effectiveness of IPE. Issues such as crowded timetables and the logistical difficulties of having large numbers of students participate in the same learning activities simultaneously are significant obstacles [16]. Despite these challenges, several accreditation bodies such as the Australian Health Practitioner Regulation Agency [17] have integrated IPE components into their standards, prompting an increasing number of healthcare education committees to consider and develop IPE within their institutions. However, there is a scarcity of literature that evaluates the impact of IPE on the development of core competencies across different healthcare professions.

The Interprofessional Education Collaborative (IPEC) [18] identified four core competencies of interprofessional collaboration. These competencies are Values and Ethics, Roles and Responsibilities, Communication, and Teams and Teamwork. These competencies enhance patient care, promote population health and reduce healthcare costs. However, the extent to which IPE sessions have been used to foster acquisition of these core competencies among healthcare students has not been investigated. Therefore, this review aimed to inductively analyse the reported competencies developed by healthcare students through IPE activities in clinical practice settings and then deductively map the identified themes and sub-themes to the IPEC framework. Additionally, the review aimed to identify the positive and negative impacts of IPE on the education outcomes for healthcare students. The following research questions are addressed in this review: a) What are the core interprofessional competencies developed through IPE programs in health professions education? b) What impact does IPE have on healthcare education outcomes and clinical practice for health professions students? This evaluation approach provides a nuanced understanding of the effectiveness of IPE, provides guidance to healthcare educators on curriculum development and highlights future research directions.

## Methods

A scoping review methodology was employed to identify and synthesise evidence on the impact of IPE activities within clinical practice settings in fostering the development of core interprofessional competencies and its positive and negative impacts on educational outcomes for healthcare students. Scoping review is used to determine the extent and coverage of literature on a given topic, providing a clear indication of the available literature and its focus [19]. Unlike systematic reviews, scoping reviews have broader inclusion criteria, allowing for a more expansive exploration of the literature [19, 20]. This scoping review followed a four-stage process: problem identification (clearly defining the research question and purpose), literature search (using a comprehensive search strategy), data evaluation (assessing methodological quality) and data analysis (synthesising findings based on inductive themes) [21].

### Inclusion criteria

The review included studies that involved undergraduate students from healthcare professions. To be considered, papers had to specifically address the impact of IPE within the context of healthcare practice and report on two or more professions working collaboratively in the healthcare environment/ setting. Only peer-reviewed original research articles published in English from 2004 to 2024 were included to capture the most up-to-date research.

### Exclusion criteria

The review excluded papers that did not focus on interprofessional education among health professions students. Studies evaluating perceptions without specific IPE activities, describing a program without any students’ outcome measured, teaching IPE in settings other than clinical practice settings (such as class discussions, workshops and simulations without any form of experiential learning), targeting interprofessional education in professions outside the healthcare environment, validating tools or focusing on non-health students were not considered. Additionally, general exclusion criteria included conference abstracts, opinion papers, book chapters, editorials, commentaries, clinical case studies, theses and review studies.

### Search strategy

Five electronic databases were selected for the search: Medline, Scopus, Web of Science, PsycINFO and EBSCO. The search terms included combinations of keywords such as "interprofessional learning", "interprofessional education", "interprofessional training", "healthcare students", "medical", "nursing", "allied health", "speech therapy", "radiography", "competency" and other related terms. Boolean operators (AND, OR) were utilised to refine and broaden the search as necessary, ensuring the inclusion of all relevant studies. The search was limited to peer-reviewed original research articles published in English. Additionally, reference lists of the selected articles were manually searched to identify any further relevant studies that may have been missed during the database search.

### Data extraction

Three primary authors (EB, FM and BMA) independently screened the titles and abstracts of all the retrieved papers against the inclusion and exclusion criteria. The same three authors independently extracted data from each study to minimise bias and ensure reliability. A standardised data extraction form was developed and used to capture relevant details from the included studies. The form included fields for characteristics of the reviewed studies including citation details, study design, participant information and summary of findings.

Additionally, competencies attained by the healthcare students in the study were categorised and mapped to the four IPEC core competencies framework [18]. Furthermore, the positive and negative impacts of IPE on healthcare students’ clinical learning outcomes were identified.

### Data synthesis

A mixed methods synthesis approach was used to analyse the extracted data. This approach involved integration of qualitative and quantitative studies into a single narrative synthesis that addresses a concept [22, 23]. This approach aided the synthesis and coding of the major outcomes from each study. Core competencies developed and outcomes were inductively coded and categorised into themes and sub-themes. Content analysis [24] was performed to determine the frequency of occurrence of each coded outcome across the included studies. This analysis provided insights into which outcomes were most common. The identified themes were subsequently deductively mapped to the IPEC framework based on framework analysis to determine the IPEC core competencies that were developed. The narrative synthesis also provided a detailed account of how IPE influenced the development of core competencies, and the challenges encountered. This categorisation allowed for a structured analysis of the impact of IPE within each study context. Discrepancies between the authors during coding and theme generation were resolved in a consensus meeting.

### Risk of bias assessment

The Quality Assessment for Diverse Studies (QuADS) was used to assess the methodological quality of the included papers. The QuADS tool demonstrates substantial interrater reliability and content and face validity and is more applicable to assess the quality of multi or mixed-methods research [25]. QuADS has a total of 13 questions, with rating criteria ranging from zero (0- not stated at all) to three (3-explicitly described) [25]. All authors were involved in this process, with three reviewers independently assessing the methodological quality of each article. To assess the methodological quality of each of the reviewed studies, the criteria scores were summed up and expressed as a percentage of the maximum possible score. The percentage scores were classified into low (< 50%), medium (50–80%) or high (> 80%) quality evidence for comparison.

## Results

### Included papers

**Fig. 1:**
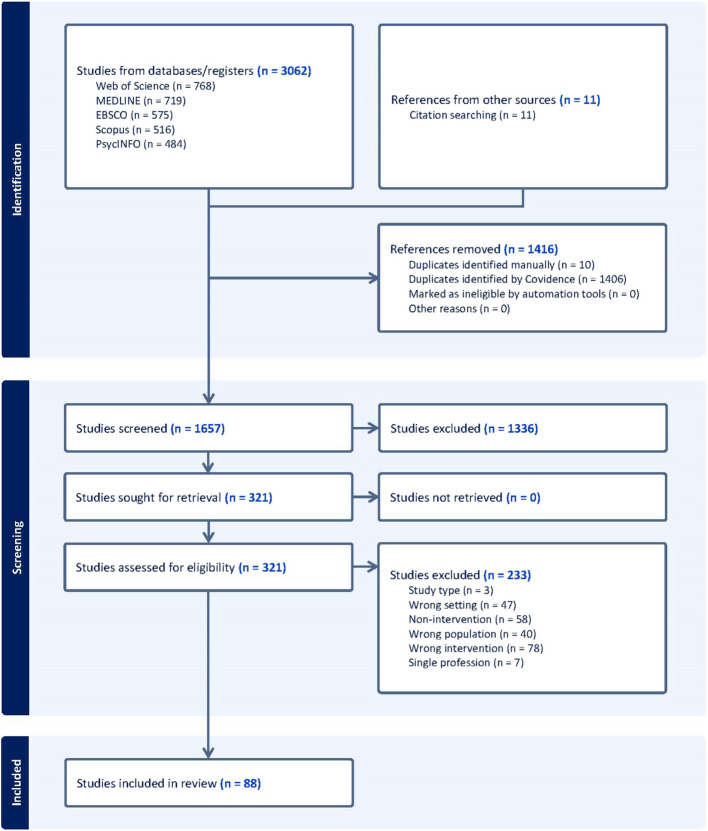
Flow chart of the study selection protocol

A total of 3073 articles were identified from all searched databases and imported into Covidence. After removing 1416 duplicates and screening the titles and abstracts of the remaining 1657 articles, 321 were identified for full text review. Eighty-eight (88) studies [12, 26–112] met the inclusion criteria for this review (Fig. 1).

### Characteristics of the included papers

**Fig. 2:**
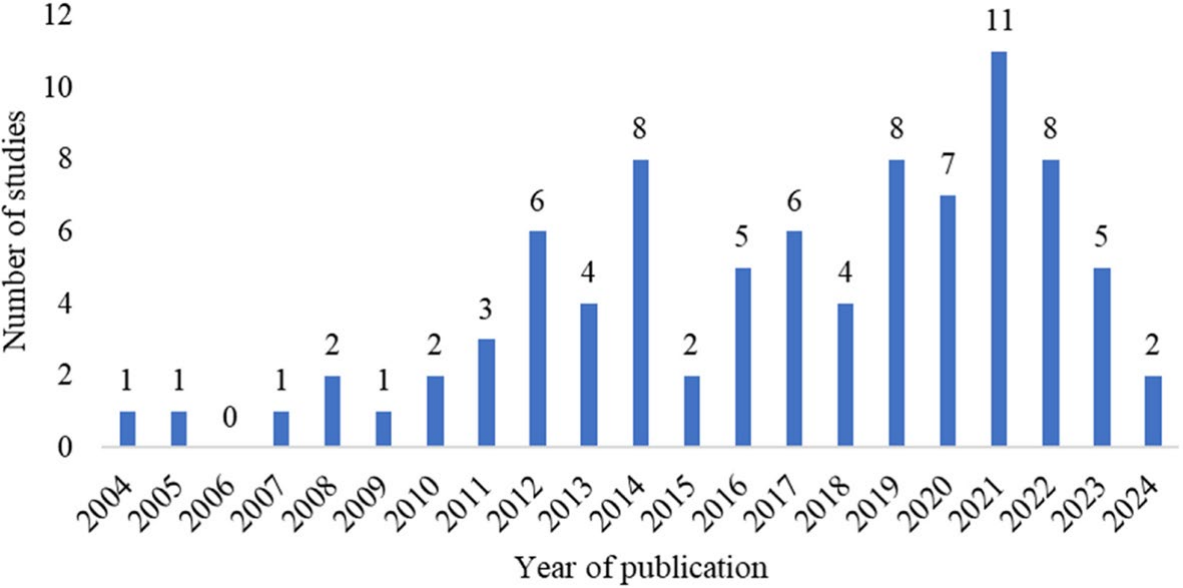
Distribution of the included studies by year of publication

**Fig. 3:**
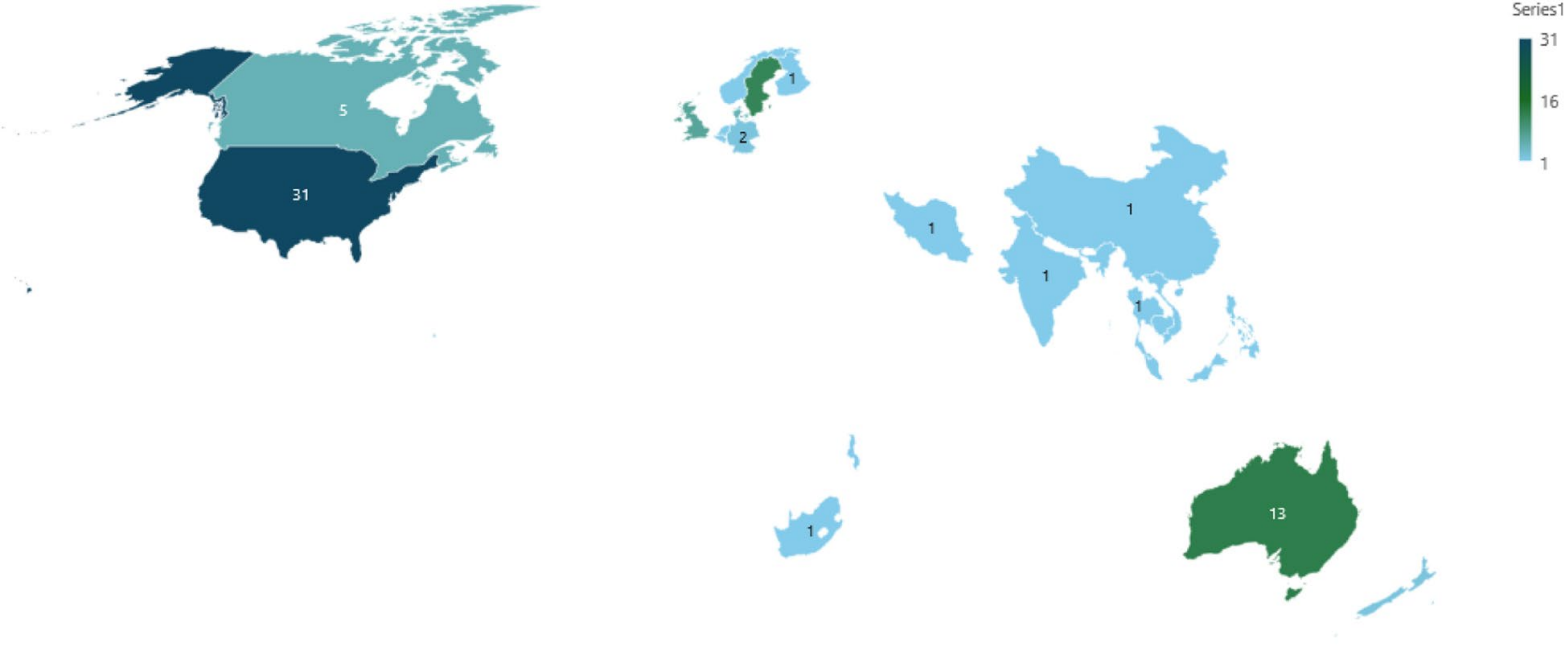
Geographic distribution of the studies included in the review

A summary of the study characteristics of the included studies is presented in Supplementary Table 1. Of the 88 included studies, 67 (76%) were published in the last 10 years (Fig. 2). The studies were conducted in a variety of hospital settings (64; 74%), community settings (21; 24%) or a combination of both (3; 2%). Thirty (34%) of the included papers were conducted in the USA, twelve (14%) in Australia, eleven (13%) in Sweden, six (7%) in the UK, five (6%) in Canada and five (6%) in Denmark. (Fig. 3). There was one study conducted in Austria, Belgium, China, Finland, India, Iran, Malaysia, Netherlands, Philippines, South Africa, Thailand, and Trinidad and Tobago, respectively, while three studies took place in multiple countries: one in Australia, Cambodia and Vietnam, another in Sweden and Norway and the third one in Malawi and USA.

**Fig. 4:**
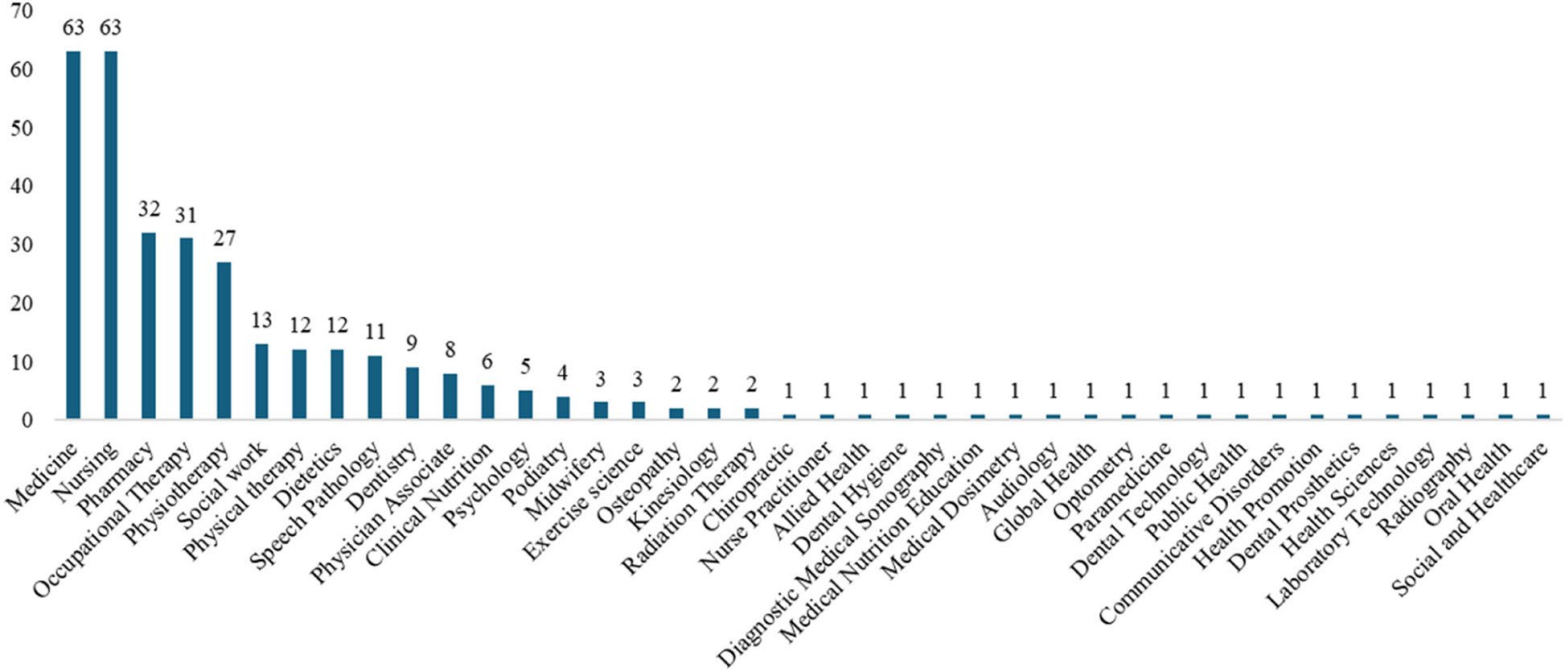
Distribution of health professions in the included studies

Majority of the reviewed studies included medical (72%) and nursing (72%) students (Fig. 4). Pharmacy (37%), occupational therapy (36%) and physiotherapy (31%) students were represented in at least a third of the studies. Twenty allied health professions were represented in only one study per profession. However, one study [26] did not specify the allied health professions of the participating students.

The majority of the included papers (44%) used mixed methods study design involving both quantitative and qualitative methods, 27 (31%) used quantitative methods alone, and 22 (25%) used qualitative methods alone (Supplementary Table 1). The studies that used quantitative methods, collected data using questionnaires and validated instruments such as the Readiness for Interprofessional Learning Scale, IPEC Competency Self-Assessment and the Nebraska Interprofessional Education Attitudes Scale, Student Perceptions of Interprofessional Clinical Education instrument, the Interprofessional Socialisation and Valuing Scale and the Interdisciplinary Education Perception Scale. The qualitative studies used interviews, focus group discussions, reflections and essays.

### Identified themes and sub-themes

Supplementary Table 2 presents the various IPE activities reported in the reviewed articles as well as the key outcomes related to core competencies and the impacts (both positive and negative) of the educational strategies employed. Inductive content analysis of the competencies developed in the included articles identified two themes and eight sub-themes explaining the core competencies developed by healthcare students during their IPE learning experiences. The two themes are personal professional growth and professional interactional skills/ competencies (Table 1). The two themes and their respective sub-themes are described below.

**Table 1:** Identified themes, sub-themes and their respective Interprofessional Education Collaborative (IPEC) core competencies

Theme	Sub-themes	IPEC core competencies	References
Personal professional growth	Collaborative learning	Teams and Teamwork	[12, 26–28, 31, 37, 38, 44, 47, 55, 57, 62–64, 67, 72, 74, 75, 80, 86, 90, 95, 110]
	Professional identity and confidence building	Values and Ethics	[27, 30, 31, 38, 39, 45, 50, 64, 65, 76, 85, 96, 103, 109]
	Clarity of roles and responsibilities	Roles and Responsibilities	[27, 31, 36, 37, 39, 42, 45, 47–49, 57–59, 71, 77, 80, 81, 84, 85, 88, 90, 93, 97, 98, 100, 104, 105, 107, 108]
	Effective communication	Communication	[26, 30, 35, 37, 43, 48, 50, 53, 55, 57, 59, 60, 62, 69, 75, 89, 91–93, 96, 98, 101, 104, 109, 111, 112]
Professional interactions for improved patient care	Shared approach to patient-centred care	Teams and Teamwork	[12, 33, 36, 45, 48, 57, 63, 72, 76, 84, 98]
	Collaborative work	Teams and Teamwork	[26, 28, 30, 31, 34, 35, 38, 42, 44, 45, 47–49, 53, 56, 57, 60, 61, 63, 69, 72, 73, 78, 81, 83, 84, 87, 89–91, 93, 98, 100–103, 106, 110]
	Support and supervision	Teams and Teamwork	[51, 67]
	Equality and respect	Values and Ethics	[28, 31, 36, 51, 55, 59, 71, 74, 90, 110]

### Personal professional growth

The first theme primarily focuses on the direct effects of IPE on students’ personal professional growth, underscoring the profound impact of IPE on personal development within professional contexts. Central to this theme is the role of IPE in enhancing collaborative learning—a fundamental aspect for developing vital professional competencies. Collaborative environments fostered by IPE initiatives are instrumental in helping students build a robust professional identity and bolster confidence. Additionally, these educational experiences enriched students’ understanding of their roles and responsibilities within multidisciplinary teams and fostered effective communication skills.

#### Collaborative learning

This sub-theme was reported in 23 studies [12, 26–28, 31, 37, 38, 44, 47, 55, 57, 62–64, 67, 72, 74, 75, 80, 86, 90, 95, 110] and it addressed the structured learning activities designed to enhance teamwork among healthcare students from different disciplines. In this context. collaborative learning environments were pivotal in teaching students how to coordinate and integrate the strengths and skills of various team members effectively. For example, Kara et al. [63] demonstrated how IPE encourages the exchange of ideas, promoting deeper understanding and cohesion among students from different healthcare backgrounds. Additionally, the study by Alderman [28] utilised hotspotting in home health as a collaborative intervention model which involved teams of students from diverse health disciplines, who conducted a total of 32 home visits. The study demonstrated a significant reduction in 30-day hospital readmission rates for patients receiving interprofessional home visits compared to a control group (p = 0.038). This learning approach not only prepared students for the complexities of real-world healthcare settings where teamwork is crucial but also solidified the foundational practices of working cohesively towards common goals.

#### Professional identity and confidence building

Focusing on the personal growth of students, this sub-theme reflects the development of a secure professional identity and ethical practices within healthcare settings. This sub-theme was presented in 14 studies [27, 30, 31, 38, 39, 45, 50, 64, 65, 76, 85, 96, 103, 109] and it emphasises the moral and ethical standards integral to healthcare professions. Through IPE, students encountered scenarios that challenged them to think critically about ethical issues and develop a strong moral compass as healthcare providers. For instance, the study conducted by Keshmiri & Barghi [65] utilised a community-based education program which enabled learners in interprofessional teams to go into the community to assess health needs and provide education on specific topics. The community environment provided the learners an authentic situation to improve their skill, attitude and self-efficacy. Another study by Omar et al. [85] illustrated how students gained confidence in their abilities and a clearer sense of their professional roles through interprofessional interactions. The engagement in interprofessional settings allowed students to witness and understand the ethical dilemmas that can arise in healthcare settings, preparing them to handle such challenges with integrity and confidence.

#### Role clarity

Another crucial element of IPE is students’ improved ability to understand roles and responsibilities of their own profession and other health professions. Twenty-nine studies [27, 31, 36, 37, 39, 42, 45, 47–49, 57–59, 71, 77, 80, 81, 84, 85, 88, 90, 93, 97, 98, 100, 104, 105, 107, 108] confirmed that students demonstrated an increasing understanding of roles and responsibilities after IPE programs. The study by Hallin et al. [48] reported an IPE course that took place in a clinical education ward setting. The course involved students from four different healthcare professions actively participating in interprofessional teamwork in a real clinical practice setting. The students performed all the medical, nursing, physiotherapy and occupational therapy work and care of the patients, with tutors initially acting as role models and then stepping back to provide supervision as the students became more independent. All student groups reported a clearer understanding of their own professional roles and those of other professions, with occupational therapy and medical students showing the most significant progress. The IPE initiative helped students understand each other’s roles and responsibilities, recognise their own roles, and appreciate the contributions of other professions in patient care. This initiative aimed to clarify and define these roles, ensuring that all team members understood their responsibilities and how their contributions fit within the broader team context. This understanding is crucial for reducing overlaps and gaps in patient care and for maximising the team’s collective capabilities. The prominence of this sub-theme in this review indicates a strong consensus on the value of role clarity in enhancing operational efficiency and teamwork in healthcare settings.

#### Effective communication

This sub-theme focuses on the development and refinement of communication skills necessary for clear, concise and respectful information exchange among healthcare professionals. Twenty-five papers reported that IPE helps achieve or improve interprofessional communication, clarity in communication and assertiveness in communicating [26, 30, 35, 37, 43, 48, 50, 53, 55, 57, 59, 60, 62, 69, 75, 89, 91–93, 96, 98, 101, 104, 109, 111]. The reviewed papers revealed that IPE equips students with the tools to articulate their thoughts clearly, listen actively, and respond appropriately in varied clinical situations, ensuring that all team members are on the same page regarding patient care and treatment plans. For example, the study by Holmes et al. [50] explored the experiences of an interprofessional group of students in a clinical IPE experience, wherein the students were involved in weekly 3-h visits to a senior housing facility to provide a variety of services to the older adult residents. The IPE program helped the students broaden their understanding of other healthcare professions, improve team-based communication and collaboration and increase their awareness of inter-disciplinary roles. Participating in the IPE program enabled the students to gain exposure to common issues affecting older adults and gingered some students’ interest in geriatrics as a future career.

### Professional interactions for improved patient care

This theme focuses on how students’ IPE experiences translate into improved healthcare practices, demonstrating the significant value of IPE in clinical practice settings. The studies showed that IPE enhanced students’ interactional skills, which are foundational to delivering high-quality healthcare. Key areas of development included shared person-centred care approaches, teamwork, support and supervision, as well as fostering an environment of equality and respect among healthcare students. These sub-themes collectively contribute to more efficient, collaborative and equitable healthcare outcomes.

#### Shared person-centred care approach

This sub-theme reflects how IPE trains healthcare students to adopt a shared approach, emphasising empathy, respect and the ethical treatment of patients. By learning together, students from different fields gain insights into the holistic needs of patients, fostering a more coordinated and compassionate approach to healthcare. Eleven studies emphasised the value of IPE in fostering a shared patient-centred care approach in healthcare [12, 33, 36, 45, 48, 57, 63, 72, 76, 84, 98]. For example, the study by Marcussen et al. [72] investigated the impact of interprofessional training on students’ readiness for interprofessional collaboration in a psychiatric ward and demonstrated that the intervention group showed significant improvements in all three subscales (Partnership/Shared decision making, Cooperation and Coordination) of the Assessment of Interprofessional Team Collaboration Scale (AITCS).

#### Collaborative work

This sub-theme stresses the importance of collaborative work in delivering high-quality healthcare. Thirty-eight (38) studies [26, 28, 30, 31, 34, 35, 38, 42, 44, 45, 47–49, 53, 56, 57, 60, 61, 63, 69, 72, 73, 78, 81, 83, 84, 87, 89–91, 93, 98, 100–103, 106, 110] made reference to collaborative teamwork. The large number of studies focused on this sub-theme indicates a critical evaluation point for IPE programs, showing that effective teamwork is both a method of learning and an outcome. For instance, the study by Venville & Andrews [106] exemplified how collaborative projects can lead to improved problem-solving skills and innovative patient care strategies. Additionally, the work done by Takahashi et al. [100] involved formal integration of IPE into the clinical practice setting in a spinal bifida clinic. The results showed that students reported increased understanding of their own and others’ roles and a more holistic view of patients and families and demonstrated their ability to work in teams to create collaborative care plans. Clinic team members enjoyed participating in the program, were impressed by the students’ collaborative work, and became more aware of interprofessional issues in their own practice. The focus here extended beyond mere collaboration to include the integration of diverse professional perspectives to enhance interactional skills and patient outcomes. Effectively working together as an interprofessional team improved perceptions of the need for teamwork, attitudes toward shared learning and positive beliefs about teamwork.

#### Support and supervision

Support and supervision are critical components of interprofessional collaborative healthcare, and IPE plays a vital role in enhancing these aspects. This sub-theme underscores the need for adequate support and supervision in clinical training environments. As reported in the reviewed articles, effective supervision ensured that educational goals were met while providing a safety net for both students and patients. This sub-theme highlights the importance of guidance and mentorship in shaping the clinical skills and professional attitudes of healthcare students. The study by Lachmann et al. [67] observed that proactive supervision helped students navigate the complexities of interprofessional interactions, providing them with guidance and feedback that is critical for their development as healthcare professionals. Another study [51] investigated the outcomes of an interprofessional clinical placement program in the emergency department of a metropolitan hospital, where final year nursing and medical students worked in pairs for 2-week placements, providing direct patient care under the supervision of trained interprofessional facilitators from nursing and medicine. The emergency department was perceived as an effective environment for learning interprofessional skills and behaviours. Students reported improvements in self-efficacy for interprofessional collaboration. However, challenges were identified in the organisation and supervision of the student teams, with a lack of consistency in approaches to supervision among professional staff.

#### Equality and respect

Equality is a fundamental principle in fostering interprofessional collaborative care, and IPE has been shown to promote this through various mechanisms. Addressing the interpersonal dynamics within healthcare teams, this sub-theme was reported in 10 studies [28, 31, 36, 51, 55, 59, 71, 74, 90, 110] as vital for fostering an environment where all professions are valued equally. Robertson et al. [90] conducted a 4-week interprofessional learning experience centred on discharge planning for patients on an inpatient medical-surgical unit. Nursing and medical students were paired into dyads and assigned patients to work on. They reported that aligning subject knowledge prior to the experience to alleviate inequity was critical in contributing to a perceived positive experience. Respect and equality were fundamental for reducing conflict and enhancing team cohesion, leading to more effective patient care. By engaging in IPE, students learned to respect each other’s roles and contributions, fostering an environment of mutual respect and equality in interprofessional collaborative healthcare delivery.

### Mapping of themes to the IPEC framework

The results of the deductive framework analysis showed that the identified themes and sub-themes aligned with the four major core competencies within the IPEC theoretical framework, and IPE significantly contributed to the development of these four major core competencies.

1. **Teams and Teamwork:** This was the most frequently developed competency. It was identified in 65 instances across the studies and emphasised the ability of students to work collaboratively within diverse healthcare teams. The 62 studies [12, 26–31, 34–38, 42, 44, 45, 47–49, 53, 55–57, 60–64, 67, 69, 72–76, 78, 80, 81, 83, 84, 86, 87, 89–91, 93, 95, 98, 99, 101–105, 107, 111] that reported on this competency demonstrated that collaborative learning environments substantially enhanced team coordination and effectiveness.
2. **Roles and Responsibilities:** Clarity in professional roles was the second most reported outcome, with 32 review articles [27, 31, 36, 37, 39, 42, 45, 47–49, 57–59, 71, 77, 80, 81, 84, 85, 88, 90, 93, 97, 98, 100, 103–110] highlighting the importance of understanding and respecting the distinct functions and contributions of different health professions within a team setting.
3. **Values and Ethics:** This competency was noted in 30 articles [12, 27, 28, 30, 31, 33, 36, 38, 39, 45, 48, 50, 51, 55, 57, 59, 63–65, 71, 73, 74, 76, 84, 85, 96, 98, 104, 110, 110]. It was developed primarily through activities that fostered professional identity and confidence and promoted a shared approach to patient-centred care.
4. **Communication:** Effective communication was emphasised as critical for ensuring clear and empathetic interactions among healthcare teams, with 25 articles [26, 30, 35, 37, 43, 48, 52, 53, 55, 57, 59, 60, 62, 69, 75, 89, 91–93, 96, 98, 101, 105, 110, 112] documenting significant improvements in this area.

### Positive and negative impact of IPE interventions

Impacts of the IPE interventions were classified into positive and negative educational outcomes (Table 2).

**Table 2:** Impacts of IPE interventions

Category	Impact	References
Positive impact	IPEC core competences	[12, 26–112]
	Improvement in self-efficacy for working in interprofessional teams	[12, 51, 52, 83]
	Improved quality of care for patients	[30, 45, 111]
	Improved patient outcomes	[28, 31, 32, 46, 53, 79, 95, 96]
	Improved profession-specific knowledge and skills	[12, 33, 39, 43, 49, 64, 88, 92, 94, 97, 99, 103–105]
	Motivated future career intent to serve vulnerable and underserved patient groups	[50, 70, 74, 80]
	Understanding the complexity of health systems	[37, 88]
	Activating emotions towards learning and reflection	[59, 67, 103]
	Understanding the unique healthcare needs of vulnerable populations	[37, 50, 64, 87]
	Fulfilled sense of autonomy	[34, 107]
Negative impact	Leaving uniprofessional teams and foregoing patient care to participate in the IPE activity	[90]
	Negative personal identity development	[45, 55]
	Perceived limited benefit learning from staff from other professions	[110]
	Negative emotions or feelings	[59, 63, 67, 103, 111]
	Hierarchy (difference in power)	[88, 107]
	Insensitivity to team members	[97, 111]

Most of the studies reported an improvement in the educational outcomes for healthcare students [26–112]. Students gained profession-specific knowledge and skills [33, 36, 39, 43, 49, 64, 88, 92, 94, 97, 99, 103–105], such as identification of medication-related problems [104], knowledge of diabetes care [94], geriatric patient care [33] and clinical reasoning skills [92]. Students had improved self-efficacy [12, 51, 52, 83], experienced a sense of fulfilled autonomy [34, 106], gained a clearer understanding of the complexity of health systems [37, 88] and improved the understanding of the unique healthcare needs of vulnerable populations such as the elderly and people living with chronic diseases [37, 50, 64, 87]. Furthermore, there was improved quality of care for patients [30, 45, 103, 111] and improved patient outcomes such as fewer 30-day hospital readmissions [28] and identification of potentially serious health conditions like cardiovascular disease and diabetes [45].

Negative impacts: Although there were many benefits associated with IPE; several negative outcomes were reported. Students reported experiencing negative emotions or feelings such as feeling intimidated or nervous [63] and stress [67]. There were instances of power imbalances [88, 107] and team members being insensitive towards their interprofessional teammates [97, 110]. Students were required to leave uniprofessional teams and forego some patient care to participate in this IPE activity [90]. Some studies reported negative personal identity development in students participating in IPE [45, 55] and perceived limited benefits of learning from staff from other professions [110].

As indicated in supplementary Table 2, participants identified several challenges to implementing IPE. These included logistical difficulties of coordinating students’ schedules, resistance from some staff members and student discomfort with interprofessional interactions [56]. Additional challenges were related to coordination and communication, patient management, program structure, limitations of the community setting [86], and the organisation and supervision of student teams [51].

### Risk of bias of included studies

The QuADS results indicated that the scores ranged from 23 to 92%. There were more medium quality studies (*n* = 72) compared to low (*n* = 13) and high-quality studies (*n* = 3). Most studies had very low scores on the criteria of theoretical or conceptual framework underpinning the research and stakeholder engagement in the research. The studies with high scores were judged to be appropriate in their statistical analyses and study designs. The risk of bias assessment results are detailed in Table 3. The primary purpose of a scoping review is to map the evidence landscape, clarifying key concepts and definitions, examining research methodologies, identifying main characteristics or factors, and analysing knowledge gaps. Consequently, no studies were excluded based on quality scores.

**Table 3:** Risk of bias assessment of the reviewed studies

*QuADS Criteria **Study (Year)**	**1**	**2**	**3**	**4**	**5**	**6**	**7**	**8**	**9**	**10**	**11**	**12**	**13**	**% Maximum score**
Aggar et al., 2020 [27]	0	3	3	3	1	3	2	1	1	3	3	0	1	62
Alderman, 2022 [28]	1	3	2	2	2	1	2	2	2	2	2	0	2	59
Anderson & Thorpe, 2010 [29]	3	1	3	2	1	0	1	1	1	0	1	0	2	41
Basran et al., 2012 [30]	0	3	3	3	2	3	3	3	1	3	3	0	2	74
Berkley-Patton et al., 2021 [31]	0	2	3	3	1	0	1	1	1	0	1	1	2	41
Bradley et al., 2023 [32]	0	3	1	3	2	3	3	3	2	3	3	0	3	74
Byerly et al., 2020 [33]	2	3	3	3	1	3	3	3	1	3	3	0	3	79
Cant et al., 2014 [34]	0	3	1	2	1	2	3	3	3	2	2	0	3	64
Caratelli et al., 2020 [35]	3	3	3	3	2	1	2	2	1	2	2	2	1	69
Craig et al., 2014 [26]	3	2	2	2	2	2	3	2	1	2	2	2	2	69
Darlow et al., 2015 [12]	0	3	3	2	2	3	3	3	2	3	3	0	3	77
Darlow et al., 2022 [36]	0	1	2	2	2	3	3	3	2	2	2	1	3	67
Dressel et al., 2017 [37]	0	2	2	2	1	1	1	1	0	1	1	1	1	36
Ericson et al., 2012 [38]	0	3	2	3	2	1	2	2	2	2	2	0	3	62
Fairchild et al., 2012 [39]	0	3	2	2	0	2	2	3	2	3	3	0	2	62
Falk et al., 2013 [40]	0	3	3	3	1	2	2	3	3	3	3	0	3	74
Fell et al., 2019 [41]	0	3	3	3	2	3	3	3	2	3	3	0	3	79
Fenn et al., 2020 [42]	0	3	3	0	3	2	2	3	2	2	2	0	2	62
Frakes et al., 2014 [43]	0	2	2	2	0	1	1	1	2	1	2	0	1	38
Friedrich et al., 2021 [44]	0	3	1	3	1	3	3	3	2	2	3	0	3	69
Grace & Coutts, 2017 [45]	0	1	1	1	0	1	1	1	0	1	1	0	1	23
Hallin & Kiessling, 2016 [46]	0	2	3	3	2	3	2	2	2	3	2	0	1	64
Hallin et al., 2009 [47]	0	2	2	3	3	3	3	3	2	2	2	0	3	72
Hallin et al., 2011 [48]	0	2	3	2	2	3	3	2	2	1	2	0	3	64
Hanson et al., 2017 [49]	0	2	1	3	1	3	3	3	3	1	2	0	3	64
Holmes et al., 2020 [50]	0	3	3	3	1	1	2	2	2	3	3	0	3	67
Hood et al., 2022 [51]	0	3	1	3	3	3	3	3	3	3	3	3	3	87
Horbal et al., 2019 [52]	0	3	2	3	2	3	3	2	2	2	2	0	3	69
Howell et al., 2021 [53]	0	3	1	3	1	3	3	3	2	3	3	0	2	69
Hylin et al., 2007 [54]	0	2	3	3	2	3	3	3	3	2	3	0	3	77
Hylin et al., 2011 [55]	0	3	2	3	2	2	3	2	2	3	3	0	3	72
Jackman et al., 2016 [56]	0	3	3	2	2	2	2	1	2	1	2	2	1	59
Jakobsen & Hansen, 2014 [57]	1	2	1	2	1	1	2	2	2	2	2	3	2	59
Jakobsen et al., 2017 [58]	0	2	1	2	1	1	2	1	2	1	2	0	3	46
Jakobsen et al., 2019 [59]	0	3	2	3	3	3	3	3	1	3	3	0	2	74
Jebara et al., 2022 [60]	2	2	2	2	1	1	2	3	2	1	1	2	1	56
Joseph et al., 2012 [61]	0	2	3	2	0	1	2	1	2	1	1	1	2	46
Kangas et al., 2021 [62]	0	3	2	2	1	2	2	2	3	3	3	0	3	67
Kara et al., 2018 [63]	0	1	1	1	1	1	1	2	2	2	2	0	2	41
Kent et al., 2014 [64]	3	2	2	2	1	1	2	2	2	1	2	2	3	64
Keshmiri & Barghi, 2021 [65]	0	2	3	2	1	1	2	2	2	2	2	0	1	51
Kloppers et al., 2022 [66]	0	2	1	2	0	2	3	3	2	1	2	0	3	54
Lachmann et al., 2013 [67]	0	3	2	3	2	3	3	3	3	3	3	0	3	79
Lavender et al., 2014 [68]	0	3	2	3	2	3	2	2	2	2	3	0	2	67
Lidskog et al., 2008 [69]	1	2	1	2	2	3	3	3	3	2	3	0	3	72
Lie et al., 2016 [70]	0	3	2	3	1	3	3	3	3	3	3	0	3	77
Luebbers et al., 2017 [71]	3	3	3	3	1	3	3	3	2	3	3	3	3	92
Marcussen et al., 2019 [72]	0	2	2	3	3	3	3	2	3	3	3	0	3	77
McGettigan & McKendree, 2015 [73]	0	3	3	3	1	3	3	3	2	2	3	0	3	74
McNair et al., 2005 [74]	0	2	2	3	2	2	2	3	3	3	2	2	3	74
Meffe et al., 2012 [75]	0	2	2	2	0	1	2	2	1	1	2	1	2	46
Miller et al., 2024 [76]	0	2	2	3	2	3	3	2	3	3	3	0	2	72
Mink et al., 2021 [77]	0	3	3	3	1	2	3	2	2	3	3	0	3	72
Monahan et al., 2018 [78]	0	2	3	3	0	2	2	2	3	2	2	0	2	59
Nagelkerk et al., 2018 [79]	0	2	3	3	2	3	3	3	3	3	3	0	3	79
Nasir et al., 2017 [80]	0	2	2	2	2	3	2	1	2	1	1	0	2	51
Naumann, Mullins, et al., 2021 [81]	0	3	2	3	1	3	3	3	2	2	2	0	2	67
Naumann, Schumacher, et al., 2021 [82]	0	2	1	2	0	1	1	1	2	1	1	0	2	36
Nørgaard et al., 2013 [83]	0	2	3	3	1	3	2	3	2	3	3	0	2	69
Nwaesei et al., 2019 [84]	0	2	2	2	1	3	3	2	2	2	3	0	2	62
Omar et al., 2021 [85]	3	3	2	3	2	2	3	3	2	1	2	0	1	69
Opina-Tan, 2013 [86]	0	3	3	3	1	2	3	3	2	1	2	0	1	62
Ostertag et al., 2022 [87]	0	2	2	3	2	3	3	3	2	2	2	0	2	67
Powers et al., 2022 [88]	0	3	2	3	1	3	2	2	2	2	3	0	3	67
Ray et al., 2021 [89]	0	3	2	3	2	2	3	3	2	3	3	0	1	69
Robertson et al., 2022 [90]	0	2	1	3	0	3	3	3	0	2	2	0	1	51
Schussel et al., 2019 [91]	0	2	1	3	1	3	3	3	2	3	3	0	3	69
Seif et al., 2014 [92]	0	3	2	3	1	3	3	3	2	2	2	0	3	69
Sevin et al., 2016 [93]	0	2	2	3	1	2	2	2	1	2	3	0	3	59
Shiyanbola et al., 2012 [94]	0	3	1	2	2	2	2	1	2	1	2	0	3	54
Shrader et al., 2023 [95]	0	2	1	2	2	3	3	3	2	2	2	0	3	64
Singla et al., 2004 [96]	0	2	2	2	1	2	2	2	2	1	2	0	2	51
Storrs et al., 2023 [97]	0	3	2	3	1	3	3	3	3	2	2	1	3	74
Suwanchatchai et al., 2024 [98]	2	2	3	3	1	3	3	3	2	3	3	0	3	79
Swinnen et al., 2021 [99]	0	3	3	3	2	3	3	3	3	1	3	0	2	74
Takahashi et al., 2010 [100]	0	3	2	2	0	1	1	2	0	1	1	2	3	46
Theodorou et al., 2018 [101]	0	2	2	2	1	2	3	2	2	3	3	2	2	67
Törnkvist & Hegefjärd, 2008 [102]	0	3	2	2	0	3	3	3	2	1	2	0	2	59
Törnkvist et al., 2023 [103]	3	3	2	3	1	3	3	3	3	2	2	0	3	79
Turrentine et al., 2016 [104]	0	1	2	2	0	2	2	1	2	1	2	0	2	44
Vaughn et al., 2014 [105]	0	2	2	2	1	2	2	2	1	1	2	0	3	51
Venville & Andrews, 2020 [106]	0	3	2	3	2	3	3	3	3	2	2	0	1	69
Visser et al., 2019 [107]	3	3	3	3	3	3	3	3	2	3	3	0	3	90
Walker et al., 2019 [108]	0	1	1	1	1	1	3	2	2	2	2	1	2	49
Wang et al., 2020 [109]	0	2	1	2	1	3	3	2	2	3	3	1	3	67
Williamson et al., 2011 [110]	0	2	1	2	1	3	2	2	2	1	2	2	2	56
Wros et al., 2023 [111]	1	2	2	2	1	3	3	3	2	2	2	0	0	59
Zelić et al., 2023 [112]	3	2	3	2	2	1	2	2	3	2	3	0	3	72

^*^Quality Assessment for Diverse Studies (QuADS) Criteria: (1) Theoretical or conceptual framework; (2) Statement of research aims/objectives; (3) description of research setting and target population; (4) study design; (5) sampling; (6) rationale for choice of data collection tool/s; (7) format and content of data collection tool; (8) data collection procedure; (9) recruitment data; (10) justification for analytic method selected; (11) method of analysis appropriate; (12) research stakeholders considered in research design or conduct; (13) strengths and limitations. 0 = no mention at all, 1 = general reference/slightly appropriate; 2 = evidence of consideration/moderately appropriate; 3 = detailed description provided/completely appropriate

## Discussion

This study’s synthesis of the outcomes from various interprofessional education (IPE) interventions highlights significant contributions to healthcare education, aligning closely with the competencies defined by the Interprofessional Education Collaborative (IPEC) [18]. The findings elucidate the depth and breadth of IPE’s impact on student preparedness for clinical practice, fostering essential skills in teamwork, communication, and ethical practice. The findings from this review indicate multifaceted impacts of IPE on healthcare students, encompassing personal professional development and professional interactional skills/ competencies as well as the associated positive and negative educational outcomes. These results align with and expand upon existing literature, demonstrating IPE’s critical role in preparing healthcare students for collaborative practice [8, 11–15].

Our analysis revealed that teamwork and collaboration are among the most frequently developed competencies through IPE. This aligns with literature suggesting that effective interprofessional collaboration improves healthcare delivery and patient safety [113]. Studies consistently demonstrate that when team members from diverse healthcare backgrounds work together, patient outcomes improve significantly [7, 114]. The teamwork competency, developed through various IPE activities, helps break down professional silos, fostering a culture of mutual respect and shared responsibility which is essential for addressing complex patient needs [2].

The clarity of roles and responsibilities was another significant outcome, highlighting IPE’s role in delineating professional boundaries and expectations. This finding is crucial as role ambiguity can lead to inefficiencies and frustration within healthcare teams [115]. By understanding the scope and limitations of each profession, students can better navigate interprofessional interactions, leading to more coordinated and effective patient care [116]. This role clarity is particularly important in complex clinical practice settings where multiple specialists are involved in patient care.

Effective communication, identified as a critical outcome of IPE, underscores the necessity of clear and open interactions among healthcare providers. As noted by O’Daniel and Rosenstein [117], poor communication is a leading factor in almost 70% of sentinel events in healthcare. Our findings support the notion that structured IPE interventions can significantly enhance communication skills, thereby potentially reducing medical errors and improving patient outcomes [118, 119].

Developing a strong professional identity and ethical grounding are equally important outcomes of IPE. As healthcare becomes increasingly collaborative, maintaining a strong sense of professional values and ethics becomes crucial [3, 120]. IPE facilitates this development by exposing students to the complexities of healthcare ethics in a controlled, educational setting, which can enhance their confidence and moral reasoning skills [4].

Despite the overwhelmingly positive impacts, several challenges were identified. These include logistical issues, resistance of students to interprofessional approaches, and the stress associated with adapting to collaborative learning environments. Challenges such as coordination difficulties due to distance of designated areas and issues with patient management such as overcrowding during sessions underscored the logistical complexities of implementing IPE in less structured settings like community health environments [86]. Additionally, students sometimes felt discomfort with interprofessional interactions, highlighting the cultural and behavioural barriers to IPE integration [56]. All these findings echo the broader literature which suggests that while IPE is conceptually advantageous, its implementation can be fraught with practical difficulties that may hinder learner experiences [16].

### Implications for practice

The findings from this study on the impacts of interprofessional education (IPE) offer several important implications for clinical practice and healthcare education. These insights are crucial for healthcare institutions and educators aiming to enhance team-based care and improve patient outcomes through more effective interprofessional collaboration.

Given the improvement in teamwork and collaboration skills through IPE, healthcare institutions should consider integrating structured team-based training programs in both educational and clinical practice settings. Such programs should clarify roles and responsibilities and foster positive team dynamics, crucial for efficient patient care. Implementing ongoing IPE workshops can further reinforce these skills among healthcare students [121]. Healthcare educators and administrators should create environments that foster the development of professional identity and confidence by incorporating ethics and values education into IPE programs. Mentorship programs pairing experienced clinicians with students and newer staff can guide them through healthcare practice complexities [122].

Community-based IPE interventions bridge the gap between theoretical knowledge and practical application by placing students in real-world healthcare settings outside the traditional hospital environment. These settings often present unique, unscripted challenges and opportunities for students to apply interprofessional collaboration in diverse settings, including underserved and rural communities. This exposure not only enhances students' learning experiences but also instils a greater sense of social responsibility and commitment to serving diverse populations [123]. Other stimulating IPE delivery methods that could be explored include (1) Integrated clinical placements that are inherently interprofessional could be established, where students from different healthcare professions are paired or grouped together in clinical practice settings under the supervision of interprofessional mentors; and (2) longitudinal capstone projects that require interprofessional teams to work on extended healthcare projects, integrating various aspects of patient care, from initial assessments through to planning and implementation of treatment strategies.

Healthcare institutions need to address the challenges and negative outcomes associated with IPE, such as logistical issues and resistance to interprofessional approaches. Providing staff and students with resources for conflict resolution, stress management, and team-building activities is essential [124]. Policymakers and educational leaders should use findings from this and similar studies to inform policy and curriculum development. Advocating for regulations that support interprofessional learning and collaborative practice and developing accreditation standards that require IPE competencies is crucial. Such alignment ensures that new healthcare professions graduates are prepared to work in diverse team-based environments [9].

#### Strengths and limitations

Strengths of our review included the diverse range of studies from various countries and the studied interventions. This diversity enhances the generalisability of the findings across different educational contexts and healthcare systems. Additionally, the use of rigorous inclusion criteria and detailed risk of bias assessment, using the QuADS tool, ensures the quality and reliability of the synthesised evidence. However, the review also has limitations. Caution should be applied in the generalisation of the findings of this review as they are limited by the search criteria. The studies included in the review employed diverse methodologies and were conducted in various contexts, ranging from metropolitan clinical practice settings to rural clinics and community-based learning environments. The heterogeneity of the included studies and lack of methodological details in some of the studies could have potentially biased the review findings. Another limitation of this review is the selection of studies written in the English language only. Additionally, the implementation of IPE programs varied significantly across the reviewed studies, including differences in duration, content and instructional methods. This variability complicates the comparison of outcomes and the identification of best practices.

## Conclusion

This scoping review synthesised evidence on the impact of IPE on healthcare students’ attainment of core competencies for healthcare delivery. The synthesis of IPE outcomes from this study confirms the multifaceted benefits of interprofessional education in preparing healthcare students for collaborative clinical practice. While challenges remain, the positive impacts on teamwork, communication, role clarity, and professional identity provide compelling evidence for the continued integration and expansion of IPE in healthcare curricula. Future research should aim to refine IPE delivery methods to mitigate the negative outcomes and explore innovative strategies to enhance the sustainability and effectiveness of interprofessional collaborations. Addressing these aspects can further solidify the role of IPE in advancing healthcare education and improving patient care outcomes across diverse healthcare settings.

## Supplementary Information

Supplementary Material 1.

Supplementary Material 2.

## Data Availability

All data generated or analysed during this study are included in this published article.

## Abbreviations

IPE: Interprofessional Education
IPEC: Interprofessional Education Collaborative
IPL: Interprofessional learning
RIPLS: Readiness for Interprofessional Learning Scale
IPP: Interprofessional Practice
NIPEAS: Nebraska Interprofessional Education Attitudes Scale
QuADS: Quality Assessment for Diverse Studies
SPICE: Student Perceptions of Interprofessional Clinical Education
WHO: World Health Organization
