# Supplementary material for: A scoping review of interprofessional education in healthcare: evaluating competency development, educational outcomes and challenges
Source: BMC Med Educ. 2025 Mar 20;25:409. doi: 10.1186/s12909-025-06969-3 (PMC11924666; doi:10.1186/s12909-025-06969-3)
Supplement: Supplementary file 1 — Supplementary Material 1. [file 12909_2025_6969_MOESM1_ESM.docx]

*Supplementary Table 1: Characteristics of included studies*

| **No** | **Title** | **Study objectives** | **Methods** | **Country** | **Participants** | **Type of data** | **IPE activity** | **Main findings** |
| --- | --- | --- | --- | --- | --- | --- | --- | --- |
|  | Interprofessional primary healthcare student placements: qualitative findings from a mixed-method evaluation (Aggar et al., 2020) | To qualitatively evaluate the experiences and perceptions of students who participated in an IPE placement program and conduct a thematic analysis of focus group interviews with the students | Qualitative | Australia, Cambodia and Vietnam | Nursing, speech pathology, podiatry and occupational therapy students | Focus group discussions | Interprofessional work-integrated learning experience where undergraduate health students from nursing, speech pathology, occupational therapy and podiatry were immersed in international (Cambodia and Vietnam) and rural Australian settings and worked in teams to deliver primary healthcare project outcomes. | Students had a positive experience with the interprofessional placement and developed collaborative competencies. Key themes included overall perceptions, scope of practice, teamwork skills, future practice and placement preparation. Students gained a better understanding of other professions and the challenges of interprofessional collaboration. |
|  | Hotspotting in home health: the impact of interprofessional student-team home visits on readmission rates of super-utilizers of the health care system (Alderman, 2022) | To examine the impact of interprofessional student-team home visits on 30-day hospital readmission rates of super-utilizers receiving interprofessional student teams’ home visits in one home health agency Investigate the relationship between the number of home visits performed by interprofessional student teams and 30-day hospital readmission rates | Quantitative | USA | 2 pharmacy, 3 public health, 3 occupational therapy, 3 nursing and one medical student | Quantitative | Interprofessional student-team home visits for patients classified as “super-utilizers” of the healthcare system. Patients in the intervention group received at least one home visit by a team of students from at least two different health professions. The students also participated in regular debriefing sessions to discuss their patient cases and reflect on their interprofessional learning experience. | Patients receiving interprofessional student-team home visits had significantly fewer 30-day hospital readmissions compared to the control group. |
|  | Learning together in practice: an interprofessional education programme to appreciate teamwork (Anderson & Thorpe, 2010) | To implement and evaluate the Leicester Model of Interprofessional Education with health and social care students in a ward-based setting and assess the impact and identify the strengths and weaknesses of this interprofessional learning approach | Qualitative | UK | 100 medical students, nursing, social work dan speech therapy students | Questionnaire | Students working in small interprofessional teams (2-5 students) to interview the patient and care team, analyse the patient’s care plan and discharge process and present their findings and recommendations to the ward team. | Students learned about related policy, patient involvement in discharge, the roles and responsibilities of team members in care delivery, the importance of effective communication, the complexity of teamworking and team decisions on discharge. Students highlighted the added benefit of learning interprofessionally, and tutors reported that they saw the value of the interprofessional learning. Clinicians had to balance clinical work and teaching, and the preparation for the program took time, particularly in identifying suitable patients and reminding ward teams of their need to engage and support the students’ learning. |
|  | The longitudinal elderly person shadowing program: outcomes from an interprofessional senior partner mentoring program (Basran et al., 2012) | To describes the evaluation of the University of Saskatchewan’s Longitudinal Elderly Person Shadowing (LEPS) project, an interprofessional senior mentoring program intended to improve health science students’ attitudes toward older adults and other health care professionals and report on whether students’ attitudes toward other health care professionals changed as a result of participating in LEPS. | Mixed methods | Canada | 141 medical, pharmacy, nutrition, nursing, social work and physical therapy students | Surveys (Polizzi’s Aging Semantic Differential and Interprofessional Education Perceptions Scale) and focus group discussions | LEPS program which involved interprofessional teams of 3-4 students from various health science programs being paired with older adult “senior partners” for 4 meetings over a fall term. | Perceptions of older adults improved significantly after participating in the LEPS program, and these changes were sustained after one year. Changes in community resources for seniors and communication with seniors were seen in students’ interprofessional attitudes. |
|  | Increasing reach of the diabetes prevention program in African American churches: project fit lessons learned in using an interprofessional student service-learning approach (Berkley-Patton et al., 2021) | To report student perspectives on their role as members of interprofessional teams leading the Diabetes Prevention Program (DPP) in African American churches. | Qualitative | USA | 95 medical, physician assistant and pharmacy students | Written course evaluations, weekly reflections and iterative discussions | DPP delivered by interprofessional teams of 3-7 students from medical, physician assistant, and pharmacy programs at the University of Missouri-Kansas City. The DPP sessions were 75 minutes long, with 45 minutes of group discussion and 30 minutes of low-intensity physical activity on weeknights. | The DPP was feasible, acceptable and led to significant weight loss among participants. Participants were highly satisfied with the program and appreciated the student coaches leading the classes. Some student coaches returned to volunteer in subsequent cohorts, providing leadership and using their experience to design their own research projects. |
|  | The interprofessional care access network (I-CAN): achieving client health outcomes by addressing social determinants in the community (Bradley et al., 2023) | To describe the development and interventions of the I-CAN model and the evaluation results, which have demonstrated substantial improvement of client outcomes in the first three years following program implementation | Mixed methods | USA | Nursing, medical, dentistry and pharmacy students | Client intake assessment | Interprofessional student teams providing weekly care coordination and addressing social determinants of health for clients over weeks and months; home visits and environmental assessments to understand the significant social determinants of health issues clients face and connecting clients to community resources and accompanying them until they can navigate independently. | The I-CAN program led to substantial improvements in health-related outcomes for clients, including reductions in emergency department visits, emergency medical service calls and hospitalisations, resulting in an estimated $224,000 in cost savings. I-CAN helped many clients improve access to health insurance, primary care and stable housing. |
|  | Fostering interprofessional geriatric patient care skills for health professions students through a nursing facility-based immersion rotation (Byerly et al., 2020) | To present an IP nursing facility rotation (IP-SNF) in which medical, pharmacy and physical therapy students provide collaborative geriatric patient care. | Mixed methods | USA | 15 medical, 12 pharmacy and 5 physical therapy students | Students’ assessments and course evaluation | A 10-day interprofessional skilled nursing facility (SNF)-based rotation (IP-SNF) for senior-level medical, pharmacy, and physical therapy students. The rotation was conducted at the San Francisco VA Medical Center’s Community Living Center (CLC), a SNF caring for veterans with complex medical and mental health conditions. | The IP-SNF rotation increased students’ self-reported confidence in geriatric patient care competencies. Students and faculty/staff provided positive feedback on the IP-SNF rotation. The IP-SNF rotation was feasible to implement and did not negatively impact the workflow of the skilled nursing facility staff. |
|  | Teaching teamwork: an evaluation of an interprofessional training ward placement for health care students (Cant et al., 2014) | To describe the interprofessional clinical placements program and report on the perceived effect on learning teamwork reported by participating students. | Mixed methods | Australia | 38 medical and nursing students | Focus group and questionnaire (Interprofessional Clinical Placement Learning Environment Inventory (ICPLEI)) | 2-week interprofessional clinical placement where final-year nursing and medical students worked in teams to manage patient care in a hospital emergency department and rehabilitation ward under the supervision of clinical educator facilitators. | Students reported improved understanding of other professional roles and increased respect for the roles of other disciplines, communication and collaboration within the interprofessional team, and felt a greater sense of autonomy and responsibility in managing patient care compared to traditional clinical placements. |
|  | Development and evaluation of an interprofessional seminar pilot course to enhance collaboration between health professions at a student-run clinic for underserved populations (Caratelli et al., 2020) | To test an interprofessional pilot course’s effectiveness and quality in meeting the four main course objectives based on the four domains of the IPEC competencies. | Mixed methods | USA | 8 dentistry, kinesiology and pharmacy | Interprofessional Collaborative Competency Attainment Survey (ICCAS) instrument, pre- and post- course surveys and course evaluation survey | Five 90-minute seminar sessions and four 4-hour clinic visits. | The ICCAS instrument showed statistically significant increases in student knowledge and abilities across the four IPEC core competency domains. The pre-post course survey showed an upward trend in reported skills and abilities, though not statistically significant. The course evaluation survey showed that students found the site visits valuable and the course increased their interest in the subject. |
|  | Evaluating the health “hubs and spokes” interprofessional placements in rural New South Wales, Australia (Craig et al., 2014) | To develop an understanding of the roles and responsibilities of other health professionals, skills in working effectively in interprofessional teams, skills in providing patient/community-centred care and an understanding of the needs of rural communities | Mixed methods | Australia | 79 medical, allied health, nursing and pharmacy students | Debriefing questionnaire, Interprofessional Education Perception Scale (IEPS), Team Performance Scale (TPS) and audience feedback | IPE program where senior health professional students from different disciplines were placed in teams to work on small, locally relevant projects in rural areas for 6 weeks. The students were released from their regular clinical placements for at least half a day per week to work in their IPE teams, which met weekly with local IPE facilitators to engage in reflective exercises and progress on their projects. | The IPL placements led to positive changes in students’ attitudes and interprofessional collaborative skills. The rural-based IPL placements were well-received by the local communities, who recognised the relevance of the student projects to local health needs. |
|  | The positive impact of interprofessional education: a controlled trial to evaluate a programme for health professional students (Darlow et al., 2015) | To evaluate if an IPE programme based around long-term conditions’ management changed dietetic, medicine, physiotherapy, and radiation therapy students’ attitudes to interprofessional teams, interprofessional learning, self-reported effectiveness as a team member and perceived ability to manage long-term conditions and evaluate if students from the two larger disciplinary groups of medicine and radiation therapy responded differentially to the programme. | Quantitative | New Zealand | 9 dietetics, 36 medical, 12 physiotherapy and 26 radiation therapy students | Attitudes Toward Health Care Teams Scale (ATHCTS), Readiness for Interprofessional Learning Scale (RIPLS), the Team Skills Scale (TSS), and the Long-Term Condition Management Scale (LTCMS). | Interactive workshop with group activities, discussions, presentations and videos, home visit with a person with long-term conditions in interdisciplinary groups and delivery of a presentation on the home visit experience | The program improved students’ attitudes towards interprofessional teams and learning and students’ self-reported effectiveness as interprofessional team members and their confidence, knowledge and ability to manage long-term conditions. |
|  | Longitudinal impact of preregistration interprofessional education on the attitudes and skills of health professionals during their early careers: a nonrandomised trial with 4-year outcomes (Darlow et al., 2022) | To assess whether a preregistration IPE program changed attitudes towards teamwork and team skills during health professionals’ final year of training and first 3 years of professional practice. | Mixed methods | New Zealand | 573 dentistry, oral health, dietetics, pharmacy, physiotherapy, medical, nursing and occupational therapy students | Survey | 5-week Tairāwhiti IPE (TIPE) immersion programme, which involves from 8 different disciplines participating in supervised clinical placements, completing collaborative tasks and living and socialising together, with teaching and learning provided by an interprofessional team across diverse town and rural settings. | Participation in the 5-week TIPE program improved health professional students’ attitudes towards healthcare teams and their self-perceived teamwork skills, and these positive effects were maintained for up to 3 years after graduation. Many TIPE graduates perceived that the program had a meaningful influence on their readiness to work in teams and the way in which they performed their healthcare roles. |
|  | Local to global: working together to meet the needs of vulnerable communities (Dressel et al., 2017) | To develop IPE experiences for students to meet the healthcare needs of vulnerable populations in Malawi and Milwaukee, enhance the health and well-being of children and families in the Westlawn community through collaborative health promotion programs and address the needs of children with disabilities living in rural Malawi through interprofessional collaboration | Quantitative | Malawi and USA | 10 nursing, occupational therapy, speech and language pathology and physical therapy students | Surveys | Interprofessional learning experiences for University of Wisconsin-Milwaukee students during a short-term study abroad program to Malawi, where they developed and presented over 38 interprofessional health education projects to communities. | The IPE curriculum resulted in a statistically significant increase in students’ understanding of three interprofessional collaborative practice core competencies: values and ethics, roles and responsibilities and teams and teamwork. The IPE experiences allowed students to gain a deeper understanding of the unique healthcare needs of vulnerable populations in both Milwaukee and Malawi, and to work collaboratively to develop and implement interprofessional health education projects to address those needs. |
|  | Interprofessional clinical training for undergraduate students in an emergency department setting (Ericson et al., 2012) | To describe the organisation and educational model of interprofessional clinical training for undergraduate students in an emergency department setting, and compare the attitudes of the participating students. | Mixed methods | Sweden | 102 nursing students, 89 medical students and 43 physiotherapy students; age 23 to 39 years | Questionnaire | 2-week interprofessional clinical training program in an emergency department setting, where teams of 2 medical, 2 nursing, and 1 physiotherapy students provide care for orthopedic patients under the supervision of clinical staff. | - The concept provided a high degree of training in interprofessional collaboration for all student categories. Medical students perceived they received more profession-specific training compared to nursing and physiotherapy students. All three student categories had a more positive attitude towards the program after the training period compared to before. |
|  | Initiation of a multidisciplinary summer studentship in palliative and supportive care in oncology (Fairchild et al., 2012) | To describe the initiation of the CCI Multidisciplinary Summer Studentship in Supportive and Palliative Care in Oncology | Mixed methods | Canada | 8 clinical nutrition, medical, occupational therapy, physiotherapy and speech–language pathology | General surveys, Interdisciplinary Education Perception Scale (IEPS), Attitudes to Health Professions Questionnaire (AHPQ) | A 6-week multidisciplinary summer studentship in palliative and supportive care in oncology at the Cross Cancer Institute: mandatory clinical time, exposure to various multidisciplinary team activities and settings, an exploratory investigation project, weekly discussion groups and a final presentation. | The program led to objective attitudinal and perceptual changes in the participating students. The students practised team process and clinical skills that are transferable to future clinical placements and post-licensure practice. |
|  | One site fits all? A student ward as a learning practice for interprofessional development (Falk et al., 2013) | To understand students’ experiences of collaboration and learning in an interprofessional training ward (IPTW) through the lens of practice theory and gain a better understanding of learning and practice in IPTW. | Mixed methods | Sweden | 454 medical, nursing, physiotherapy and occupational therapy students | Questionnaire | The IPE activity involved IPTW at the Faculty of Health Sciences, Linköping University where final-year students from medicine, nursing, physiotherapy and occupational therapy programs collaborated in teams for two weeks to handle all care aspects, including personal hygiene support and their professional responsibilities under the guidance of supervisors from different disciplines. | The IPTW created a clash between students’ “expected” professional responsibilities and the “unexpected” responsibilities of caring work. The proximity between students from different professions on the IPTW opens up contexts for negotiations and boundary work. The practice architectures of the IPTW, such as the organisation of ward rounds and the requirement for all students to be present, can enable or constrain students’ enactment of their expected professional responsibilities. |
|  | Mixed methods study: a one-week international service project enhances healthcare competencies (Fell et al., 2019) | To improve healthcare students’ self-reported competencies in the interprofessional education collaborative’s core competency domains of values, ethics and roles and responsibilities and to improve healthcare students’ cultural competencies so that the healthcare needs of diverse patient populations can be met | Mixed methods | Trinidad | 26 physician’s assistant, physical therapy, nursing, audiology, occupational therapy and medical students | Surveys and Focus group discussions (Transcultural Self-Efficacy Tool (TSET) and Interprofessional Education Core Competencies (IPECC)) | 7-day international service learning experience in Trinidad for healthcare students from the University of South Alabama, where the students worked together in interprofessional teams to provide healthcare services to underserved communities in Trinidad. The intervention involved setting up clinics, providing medical screenings and specialty care and participating in daily debriefing sessions and journaling. | The clinical experience improved the healthcare students’ self-reported competencies in the IPEC core competency domains of values/ethics and roles/responsibilities, and cultural competencies. Students rated themselves highly on the pre-trip survey, leaving little room for improvement. |
|  | Interprofessional education for complex neurological cases (Fenn et al., 2020) | Evaluate the effects of a 4-session IPE training program for students across 9 different health professions Evaluate the feasibility and effectiveness of the IPE program in improving student attitudes and skills for interprofessional teamwork Replicate findings from other IPE programs showing improvements in attitudes, skills and readiness for interprofessional practice | Mixed methods | USA | 4 communicative disorders, 2 medical, 9 nursing, 6 nutrition, 1 occupational therapy, 8 pharmacy, 6 physical therapy, 4 physician assistant and 6 psychology | Attitudes Toward Health Care Teams Scale (ATHCTS), Team Skills Scale (TSS), Readiness for Interprofessional Learning Scale (RIPLS) | Four-session IPE training program for healthcare students. The program involved placing participants into interprofessional teams of 6-9 students to work on assessing a patient with a neurological condition, developing a treatment plan and presenting their plan to the larger group. | Participants expressed more positive attitudes toward team collaboration and demonstrated higher skill mastery to function within healthcare teams following program completion. |
|  | Experiences from an interprofessional student-assisted chronic disease clinic (Frakes et al., 2014) | To develop future health workforce trainees to attain appropriate levels of interprofessional capacity | Mixed methods | Australia | 47 exercise physiology, nutrition and dietetics, occupational therapy, pharmacy, podiatry, social work; majority of students were aged 20–24 and female | Interviews | Student placements of 2-10 weeks duration for students in various allied health disciplines. Students spend 70% of their time in profession-specific activities and 30% in interprofessional team activities (intake clinics, case conferences, communication) | The students reported significant improvements in their discipline-specific knowledge, understanding of interprofessional practice and communication skills as a result of their placements at the CAHP clinic. A very high proportion of students (98%) said they would recommend the CAHP placement to other students, indicating a very positive overall experience. |
|  | SIESTA: a quick interprofessional learning activity fostering collaboration and communication between paediatric nursing trainees and medical students (Friedrich et al., 2021) | To identify factors that made participants learn from, with and about each other during SIESTA sessions. | Mixed methods | Germany | 13 medical students and 13 paediatric nursing trainees | Questionnaire and focus group interviews | SIESTA learning activity, which involved paediatric nursing trainees and medical students participated in a total of four 30-minute peer teaching sessions, supervised by registered nurses and physicians. The SIESTA sessions were part of a larger interprofessional training ward (IPAPAED) that the participants were enrolled in. | SIESTA sessions promoted interprofessional communication and self-perceived understanding of interprofessional roles and tasks for both paediatric nursing trainees and medical students. Participants reported at least moderate learning gains from the SIESTA sessions and highlighted the workplace-based nature and peer-learning aspects as key factors that fostered interprofessional learning. |
|  | An interprofessional health assessment program in rural amateur sport (Grace & Coutts, 2017) | To facilitate interprofessional learning (IPL) of students at a rural university through a multisectoral health assessment program and promote health in players of rural amateur sport. | Mixed methods | Australia | 8 nursing, 16 osteopathy and 2 exercise science students | Interprofessional learning scale (RIPLS) questionnaire and focus group discussions | A multi-component interprofessional health assessment program for rural amateur rugby league players and their families, coaches, and managers. The program involved three rounds of health assessments conducted over 3 months leading up to the rugby league season. The assessments were conducted by general medical practitioners, nurse practitioners, nursing students, osteopathy students, and exercise science students, and included patient history, profession-specific assessments, screening for serious conditions, and follow-up assessments and referrals as needed. | The health assessments identified potentially serious health conditions like cardiovascular disease and diabetes that required further medical attention and led to recommendations for training modifications to improve player health and performance. The interprofessional learning experience was valued by the students. |
|  | Effects of interprofessional education on patient perceived quality of care (Hallin et al., 2011) | To assess the patients’ perceptions of collaborative and communicative aspects of care when treated at a clinical education ward (CEW) as compared to usual care | Quantitative | Sweden | Medical, nurse, physiotherapy and occupational therapy students | Questionnaire | The IPE activity in this study was the treatment of patients by supervised interprofessional student teams at a CEW for a 2-week mandatory course. | Patients treated by interprofessional student teams at the CEW perceived higher quality of care compared to patients treated at the regular ward. CEW patients reported higher levels of participation in treatment decisions, satisfaction with information about home assistance and feeling that their home situation was taken into account during discharge planning and felt better informed overall. |
|  | A safe place with space for learning: experiences from an interprofessional training ward (Hallin & Kiessling, 2016) | To explore medical, nurse, physiotherapy and occupational therapy students’ perspectives on the process of their own learning in the sociocultural context of an interprofessional training ward (IPTW) | Qualitative | Sweden | 333 students (159 nurse, 98 medical, 45 physiotherapy and 31 occupational therapy) | Course evaluation questionnaire | IPTW which was a 2-week mandatory course for medical, nursing, physiotherapy and occupational therapy students. The students worked in interprofessional teams to provide care for patients at an authentic hospital ward, supervised by an interprofessional staff. | A safe, supportive and permissive learning environment at an IPTW enabled students to develop personally, professionally and interprofessionally and obtain motivation and faith in themselves as future healthcare professionals. If the learning environment is impaired, students’ development may be limited to only personal and/or professional development, lacking interprofessional competence and a comprehensive view of practice. |
|  | Active interprofessional education in a patient based setting increases perceived collaborative and professional competence (Hallin et al., 2009) | To evaluate whether students perceived they had achieved interprofessional competence after participating in patient-based teamwork training during an interprofessional clinical education ward (CEW) course, and to assess changes over time in students’ perceived knowledge and understanding of their own and other team members’ professional roles, as well as their comprehension of communication and teamwork collaboration skills in patient care | Quantitative | Sweden | 616 undergraduate students from medical (175), nursing (290), physiotherapy (83), and occupational therapy (68) programs | Questionnaires | An IPE course that took place in a clinical education ward (CEW) setting. The course involved students from four different healthcare professions actively participating in interprofessional teamwork in a real clinical setting. The students performed all the medical, nursing, physiotherapy and occupational therapy work and care of the patients, with tutors initially acting as role models and then stepping back to provide supervision as the students became more independent. | All student groups perceived that they had achieved the interprofessional learning objectives of the CEW course. Occupational therapy and medical students had the greatest achievements. All student groups perceived increased clarity in their own professional role as well as the roles of the other professions. |
|  | Following the growth of sarah’s baby: an interprofessional education activity for medical nutrition education and diagnostic medical sonography students (Hanson et al., 2017) | To develop student appreciation for the benefits of interprofessional teamwork, demonstrate an understanding of the assessment of fetal growth and nutrients requirements during pregnancy | Mixed methods | USA | 9 Diagnostic Medical Sonography (DMS) and 6 Medical Nutrition Education (MNE) students | Survey and debriefing, observation during the activity, and a pre and post-test | Discipline-specific online library modules using a case-based scenario to focus on the use of library resources and evaluation of foetal growth, hands-on activity where DMS students led activities to educate MNE students on assessing foetal growth, including presentations, interactive stations and scanning a phantom and patient, and a group education session led by MNE students on nutrition during pregnancy, including researching nutrient requirements and food sources. | The interprofessional education activity resulted in a statistically significant improvement in combined test scores of DMS and MNE students, both DMS and MNE student groups showed statistically significant improvements in their individual test scores. Students had a positive attitude towards the interprofessional education activity, with no students disagreeing with any of the statements about interprofessional education. |
|  | Students’ perceptions of interprofessional education in geriatrics: a qualitative analysis (Holmes et al., 2020) | To explore the experiences of an interprofessional group of students in a clinical IPE experience with older adults | Qualitative | USA | 8 pharmacy, 6 nursing, 5 social work and 4 medical students | Students’ reflective journals | Weekly 3-hour visits by an interprofessional team of students to a senior housing facility to provide a variety of services to the older adult residents. | The IPE program helped students broaden their understanding of other healthcare professions, improve team-based communication and collaboration and increase their awareness of disciplinary roles. Participating in the IPE program enabled students to gain exposure to common issues affecting older adults and affected some students’ interest in geriatrics as a future career. |
|  | Evaluation of interprofessional student teams in the emergency department: opportunities and challenges (Hood et al., 2022) | To investigate the outcomes of an interprofessional clinical placement program in the emergency department (ED) | Mixed methods | Australia | 26 nursing and 29 medical students | Survey with Self-efficacy for Interprofessional Experiential Learning Scale, the Interprofessional Clinical Placement Learning Inventory and open ended questions | an interprofessional clinical placement program in the ED of a metropolitan hospital, where final year nursing and medical students worked in pairs for 2-week placements, providing direct patient care under the supervision of trained interprofessional facilitators from nursing and medicine. | The ED was perceived as an effective environment for learning interprofessional skills and behaviours. Students reported improvements in self-efficacy for interprofessional collaboration after the placement. Challenges were identified in the organisation and supervision of the student teams, with a lack of consistency in approaches to supervision among professional staff. |
|  | Outcomes from a single-intervention trial to improve interprofessional practice behaviors at a student-led free clinic (Horbal et al., 2019) | To determine if interprofessional collaboration (IPC) can be modified through an intervention and to test the effect of an educational intervention designed to increase knowledge about partner roles and scope of practice | Quantitative | USA | 334 pharmacy, medical, social work, occupational therapy, physical therapy, law, dentistry, global health, social work, optometry and nursing students; 112 male and 222 female | Survey (Interprofessional Socialization and Valuing Scale) | 15-minute educational session that covered the interprofessional structure of the clinic, a screening tool, key points about the different partners and the process for making referrals to other partners as the pre-clinic day intervention. | The intervention group had significantly higher frequencies of correctly identifying the roles of nursing, law, dental and global health disciplines compared to the control group. The intervention group had higher self-reported favourability, indicating they valued working with others, were willing to work with others and believed in their ability to work with other healthcare professionals. |
|  | “I learned that i am loved”: older adults and undergraduate students mutually benefit from an interprofessional service-learning health promotion program (Howell et al., 2021) | To develop and implement a service-learning interprofessional health promotion program for older adults, integrate the program into university course curricula to meet gerontology competencies and evaluate the program’s outcomes, limitations and next steps for improvement | Mixed methods | USA | 25 undergraduate students (ages 22–35) in health sciences, kinesiology, and dietetics and nutrition and 14 older residents (aged 50+ years) of the housing community. | Survey and reflection papers | a 10-week health promotion program called “Community Wellness Breaks (CWB)” that was delivered by teams of 4-6 undergraduate students from different disciplines (population health sciences, kinesiology, and dietetics/nutrition). Each weekly 1-hour session included at least 30 minutes of light physical activity and educational content on topics like nutrition, physical activity, and stress reduction for older adult residents (aged 50+ years) of the housing community. | The program had positive outcomes for both the undergraduate student participants and the older adult participants, including increased student learning and comfort working with seniors, and positive health and psychological outcomes for the older adults. The older adult participants reported high levels of satisfaction with the CWB program. The CWB program influenced some students to consider careers working with older adults. |
|  | Students’ approaches to learning in clinical interprofessional context (Hylin et al., 2011) | To categorise the students’ learning approaches by using the Conceptions of Knowledge and Learning Questionnaire and to relate these to their professions, gender, attitudes towards the course and the course goals, understanding of their own professional role, knowledge of the other professions and their satisfaction with the supervision. | Quantitative | Sweden | 60 medical, 151 nursing, 33 occupational therapy and 39 physical therapy; 43 male and 238 female | Questionnaire including the Conceptions of Learning and Knowledge Questionnaire (CLKQ) | A two-week mandatory IPE course where three student teams work together and provide the patients with medical care, nursing care and rehabilitation supervised by profession specific facilitators. | Students with a low collaborative approach to learning were less satisfied with the interprofessional clinical training course and found the goals less important compared to other students. Students with a “cookbook” approach, focused on practical and certain knowledge and showed an increased understanding of the importance of interprofessional collaboration after the course. |
|  | Interprofessional training in clinical practice on a training ward for healthcare students: a two-year follow-up (Hylin et al., 2007) | To examine former students’ impressions of the interprofessional course they had taken as undergraduates and examine the relationship between the interprofessional course and the former students’ current healthcare experiences, including their use of the interprofessional skills they learned | Mixed methods | Sweden | 348 Nursing, medical, physiotherapy and occupational therapy students | Questionnaire | A two-week mandatory interprofessional training course involving students working in interprofessional teams of 5-6 students. | The majority of former students reported encouraging teamwork in their current occupations, suggesting a lasting impact of the training. Experts recommend that interprofessional education should be introduced early in healthcare education programs to promote collaboration and understanding between different professions. |
|  | A rural interprofessional educational initiative: what success looks like (Jackman et al., 2016) | To investigate the psychosocial processes underlying an interprofessional preceptorship pilot for nursing and medical students in a rural health care setting and to promote interprofessional preceptorship development through evidence-based, qualitative findings | Qualitative | Canada | 3 female nursing students aged 21-23 years. 4 medical students (1 male, 3 female) aged 24-29 years. | Semi-structured interviews and focus group discussions | IPE pilot program where nursing and medical students undertook concurrent preceptorships in a semi-rural acute care setting, with the goal of emphasising interprofessional collaboration and team-building. The duration of the intervention ranged from 2 weeks to 10 weeks, depending on the student. | The rural IPE initiative led to enhanced knowledge of each other’s roles, increased confidence in interprofessional collaboration and students emulating the interprofessional collegiality of their rural preceptors. The success of the IPE program depended on support and facilitation from preceptors and staff, as well as proactive engagement from the students. Barriers to success included logistical challenges, resistance from some staff, and student discomfort with interprofessional interactions. |
|  | Spreading the concept: an attempt to translate an interprofessional clinical placement across a Danish hospital (Jakobsen & Hansen, 2014) | To evaluate whether the students had the opportunity to work as an interprofessional team with the patient as common ground and with daily team meetings, tutor challenge and support, and training in independent action and decision making. | Qualitative | Denmark | 6 nursing, 7 physiotherapy and 4 occupational therapy students | Group interviews | A one-week interprofessional clinical placement where students are responsible for patient’s care and rehabilitation in a Danish Interprofessional Training Unit | The placement contributed to students learning about interprofessional collaboration and to their development of professional identity. Clinical tutors need to create a safe and challenging learning environment, but thorough planning and continuous monitoring and adjusting of the clinical placement are necessary for success. |
|  | The interprofessional learning experience: findings from a qualitative study based in an outpatient setting (Jakobsen et al., 2017) | To understand the authentic interprofessional learning experience of students in an outpatient clinic setting | Qualitative | Denmark | 4 nursing students and 13 medical students | Interviews | Pairing one medical student and one nursing student to examine and treat patients in a dedicated examination room for 3 hours on two weekdays. | The students had a positive learning experience characterised by direct patient contact, interprofessional collaboration and task-based learning. The supervisors and management worked together to create a safe and challenging learning environment for the students. The students preferred indirect supervision that allowed them to have direct communication with patients, rather than having supervisors present during the consultations. |
|  | Emotions and clinical learning in an interprofessional outpatient clinic: a focused ethnographic study (Jakobsen et al., 2019) | To investigate the self-reported and observed relationship of the four types of emotions (activity, outcome, epistemic, and social) in the interprofessional clinical context and to analyse the valence and activation of these emotions and their role in medical and nursing students’ learning in a clinical interprofessional context. | Qualitative | Denmark | Medical and nursing students | Interviews and observational notes | Medical and nursing students’ uniprofessional clinical placements were supplemented with an interprofessional initiative on two days per week examining and caring for outpatients in a busy orthopaedic outpatient clinic. | The study identified two main themes related to student learning in the interprofessional clinical setting: “self-regulated learning” and “cooperative learning”. Students primarily experienced activating emotions, both positive and negative, rather than deactivating emotions, and that negative emotions could lead to positive emotions during the reflection phase. A safe and challenging learning environment, with appropriate supervision and support, in promoting positive emotions and learning among the interprofessional student teams are important. |
|  | Pharmacy and medical student interprofessional education placement week (Jebara et al., 2022) | To describe and present an initial evaluation of an IPE experiential learning clinical placement for pharmacy and medical students. | Qualitative | UK | 5 pharmacy and 4 medical students | Focus group discussions | 5-day IPE placement programme students worked in pairs to produce PowerPoint presentations on clinical therapeutic management based on case studies. Students completed mini-CEX assessments in their pairs, taking a clinical history from a patient and a ward-based simulation where students acted as junior pharmacists and doctors | The IPE placement was well perceived by students and improved their learning experience and understanding of each other’s roles. The placement helped prepare students for more collaborative practice in the future. Students expressed a desire to change the clinical area of the placement to allow both students to gain the most from the experience. |
|  | Interprofessional education in practice (Joseph et al., 2012) | To ascertain attitudinal change experienced by healthcare students undertaking IPE activities in clinical practice. | Quantitative | UK | 18 Medical, 12 nursing, 1 Occupational therapy and 4 pharmacy students. 1 Postgraduate social worker student | RIPLS Questionnaire | IPE activities focused on patient safety in the perioperative journey, including surgical briefing, surgical pause and recognising each profession’s contribution to patient safety, and IPE activities where students familiarised themselves with a patient from the GP caseload, considered their own profession’s perspective on the patient’s care, and presented their findings in a seminar. | IPE activities in clinical practice settings had a positive effect on students’ perceptions of interprofessional working, hence, participating in IPE during clinical placements can lead to positive attitudinal changes in students towards interprofessional working. |
|  | Students’ perceptions of interprofessional collaboration on the care of diabetes: a qualitative study (Kangas et al., 2021) | To explore changes in medical and nursing students’ perceptions of IPC on diabetes management after an experimental, voluntary course of IPE with practical content | Qualitative | Finland | 30 medical and nursing students | Focus group discussions | Interactive lectures, small group discussions and nurse-physician pair visits to a gerontological ward and diabetes outpatient clinic to foster teamwork and interprofessional learning | The course improved the students’ self-perceived abilities and confidence to perform interprofessional collaboration on diabetes care, and changed the students’ understanding of interprofessional collaboration towards a more patient-centred and holistic perspective. |
|  | An interprofessional patient assessment involving medical and nursing students: a qualitative study (Kara et al., 2018) | Explore the perceptions and experiences of nursing and medical students participating in IPE activity involving a joint patient assessment Gain insights that could inform the design of future IPE activities and improve patient care through better interprofessional collaboration | Qualitative | USA | 21 nursing and 21 medical students; 15 male and 27 female, 23 - 56 years | Debriefing interview | Interprofessional education activity in an acute care setting | Students initially felt apprehensive and intimidated about the interprofessional activity, but were able to overcome these feelings and recognise the value of their partner’s approach. Students became more aware of their own assessment deficits and the importance of teamwork in providing optimal patient care, and expressed a desire to participate in more interprofessional educational experiences. |
|  | The mixed-discipline aged-care student clinic: an authentic interprofessional learning initiative (Kent et al., 2014) | To report the process of developing an interprofessional aged-care student clinic, student learning outcomes from participating in an interprofessional aged-care student clinic and student and educator feedback on, and satisfaction with, an interprofessional aged-care student placement. | Qualitative | Australia | 42 dietetics, medical, nursing, occupational therapy, pharmacy, physiotherapy, podiatry, social work and speech pathology | Focus group sessions | An interprofessional aged-care student clinic that involved students from multiple disciplines working together to provide health screening, identify patient care needs and make referrals for older patients recently discharged from the hospital. | Students reported developing an expanded perspective of issues that affect the health of older people, felt that they gained knowledge about the practical roles and referral pathways of other disciplines, and described enhanced interprofessional communication skills. Educators reported that they observed the students’ development of communication and referral skills. |
|  | Interprofessional education in a community-based setting: an opportunity for interprofessional learning and collaboration (Keshmiri & Barghi, 2021) | To assess the effect of interprofessional community‑based education on attitudes and performances of learners toward interprofessional collaboration and their readiness for interprofessional learning. | Quantitative | Iran | 19 medical, 4 pharmacy, 38 nursing, 32 midwifery, 12 public health and 16 nutrition students | Questionnaires | Interprofessional community-based education program that consisted of two steps: Familiarising learners with concepts of interprofessional collaboration, teamwork and specific medical content through small group discussions, and learners in interprofessional teams going into the community to assess health needs and provide education on specific topics. | The scores of learners in readiness for interprofessional learning, attitudes to interprofessional teamwork and interprofessional collaboration improved significantly after the educational interventions |
|  | The lived experience of health sciences students’ participation in an interprofessional community-based stroke class (Kloppers et al., 2022) | To explore undergraduate health sciences students’ experience of being involved in community-based interprofessional circuit-based group therapy. | Qualitative | South Africa | 12 physiotherapy, occupational therapy and speech therapy students | Semi-structured interviews | An interprofessional functional circuit-group activity (IFCGA) for clients with stroke, planned and executed by final-year undergraduate health sciences students. | The program had a positive impact on students’ collaborative competencies, including their knowledge, attitudes, skills and behaviours. Students recognised the value of interprofessional collaboration and the unique contributions of different professions in providing holistic, person-centred care for patients. |
|  | Capturing students’ learning experiences and academic emotions at an interprofessional training ward (Lachmann et al., 2013) | To investigate students’ learning experiences and academic emotions in relation to collaborative and trialogical activities during interprofessional training ward (IPTW) courses and to use the contextual activity sampling system (CASS) methodology to follow the participants’ learning activities in the actual context. | Quantitative | Sweden | 37 medical, nursing, occupational therapy and physiotherapy students; 10 male and 27 female, 22 to 50 years | Contextual activity sampling system (CASS) questionnaire | Interprofessional training ward (IPTW) course | When students viewed the activities they were engaged in as “trialogical” (involving collaborative creation of knowledge), they also reported experiencing “flow” (high challenge in combination with high competence). A high trialogical level of activities was related to students’ positive academic emotions and their feeling of the importance of the ongoing activity. There was no relationship between trialogical activity level and negative academic emotions including stress, though stress levels were higher on the first day of the course. |
|  | Fostering a culture of interprofessional education for radiation therapy and medical dosimetry students (Lavender et al., 2014) | To provide an overview of a vertical interprofessional education model for radiation therapy and medical dosimetry students and to assess the effect of this interprofessional education initiative on student knowledge and communication | Quantitative | USA | Radiation therapy and medical dosimetry students | Students’ scores on end-of-training examinations and survey | 3 times per week educational conferences focused on medical residents, monthly morbidity and mortality conferences,monthly quality assurance conferences, daily peer-review sessions, daily departmental huddles, weekly chart rounds and additional optional conferences on radiobiology, physics and research | Students who participated in the interprofessional education initiative were more comfortable communicating with attending physicians, residents, physicists and faculty compared to students who did not participate. |
|  | Students’ learning experiences from interprofessional collaboration on a training ward in municipal care (Lidskog et al., 2008) | To compare students’ attitudes towards practice on a training ward before and after and to evaluate goal fulfilment after 3 weeks’ interprofessional education on a training ward. | Mixed methods | Sweden | 22 occupational therapy, 38 nursing and 5 social work; 21 to 50 years old, 5 male and 60 female | Questionnaire | An interprofessional training ward in municipal care for older people, where students from different programs worked together on the ward for 3 weeks, engaging in both profession-specific tasks and basic patient care, and sharing responsibility with permanent staff. | Students had a positive attitude towards interprofessional training on the ward both before and after the experience. Students reported greater understanding of other professions and increased motivation to collaborate after the interprofessional training. |
|  | What and how do students learn in an interprofessional student-run clinic? An educational framework for team-based care (Lie et al., 2016) | To explore both the content and process of learning, and use grounded theory to derive a framework for how the student-run clinic (SRC)-IPE experience prepares students for future team-based collaborative practice | Qualitative | USA | 50 medical, occupational therapy, pharmacy and physician assistant students | Focus group discussions | Student-run primary care clinic: an educational volunteer service activity | Participation in the interprofessional student-run clinic taught students about the roles and scopes of practice of other healthcare professions and the benefits of team-based care. The SRC experience led students to consider careers serving underserved populations and primary care. The process of learning in the SRC involves a continuum from pre-encounter team huddles to patient encounters and informal socialisation, with direct observation of other professions being a key contributor to learning. |
|  | Implementation and evaluation of a community-based interprofessional learning activity (Luebbers et al., 2017) | To implement and sustain a meaningful community-based interprofessional learning activity for large numbers of pre-licensure nursing and medical students, evaluate the barriers and facilitators of the implementation process, monitor implementation and use data-driven continuous improvement, evaluate the outcomes, including student learning outcomes and program outcomes. | Mixed methods | USA | 177 medical and 154 nursing students | Course evaluation survey and Interprofessional Collaborative Competency Attainment Survey (ICCAS) | Medical and nursing students participating in community-based screening clinics in public schools to screen for hypertension and obesity in children. | The Ottawa Model of Research Use (OMRU) framework was a useful guide for implementing, evaluating and sustaining the interprofessional learning activity. The three-step OMRU process led to important insights that improved the activity, including faculty development, role clarification and making the activity more relevant for students. All the ICCAS scores improved after the program. |
|  | Interprofessional clinical training in mental health improves students’ readiness for interprofessional collaboration: a nonrandomized intervention study (Marcussen et al., 2019) | To investigate the impact of interprofessional training on students’ readiness for interprofessional collaboration in a psychiatric ward | Quantitative | Denmark | 161 nursing, physiotherapy, social and healthcare, pedagogy and social work students | Readiness for Interprofessional learning scale (RIPLS), Assessment of Interprofessional Team Collaboration Scale (AITCS) | An initial workshop with the entire staff, weekly interprofessional group tutorial sessions where students prepared and presented on patient cases, focusing on the patient’s perspective and suggestions for interprofessional improvements and clinical care teamwork with students from different disciplines supervised by instructors. | The program improved students’ self-reported readiness for interprofessional learning and team collaboration, compared to students receiving conventional clinical training. The intervention group showed significant improvements in the “Teamwork and Collaboration” and “Positive Professional Identity” subscales of the RIPLS. The intervention group also showed significant improvements in all three subscales of the AITCS (Partnership/Shared decision making, Cooperation and Coordination). |
|  | Interprofessional training for final year healthcare students: a mixed methods evaluation of the impact on ward staff and students of a two-week placement and of factors affecting sustainability (McGettigan & McKendree, 2015) | To examine the effects on work demands and quality of care as perceived by staff, assess standard measures of ward functioning and determine whether the experience influenced students’ attitudes toward interprofessional working | Mixed methods | UK | 52 medical, nursing, physiotherapy nutrition, occupational and speech therapy students | Surveys, questionnaire and focus group discussions | The IPE activity was a two-week interprofessional training placement for final year medical, nursing, and therapy students on a rehabilitation ward at Goole and District Hospital. The ward staff were trained as facilitators for the placements, and the students worked in small groups on a 7-day-a-week schedule, being present on the ward for around 14 hours per day over the two-week period. This occurred for 7 months of the year, with a one-week break every 6 weeks. | The placement had a positive impact on students, with significant improvements in their attitudes towards teamwork, professional identity and patient-centred care. |
|  | Australian evidence for interprofessional education contributing to effective teamwork preparation and interest in rural practice (McNair et al., 2005) | To develop appropriate skills, knowledge and attitudes related to effective interprofessional practice and enhance interest in further experience in rural health care | Quantitative | Australia | 40 nursing, 40 medical, 6 pharmacy and 5 physiotherapy students; 26 male and 65 female, 23.11 years mean age | Questionnaires | A two-week interprofessional education (IPE) placement in rural Victoria, Australia for undergraduate nursing, medical, pharmacy, and physiotherapy students. The placement involved observation, collaborative projects, and online discussions, and was led by an interprofessional steering group. | The RIPE placement experience improved students’ self-reported teamwork skills and knowledge, and supported their belief in the value of interprofessional practice and strengthened nursing and allied health students’ intention to work in rural health settings after graduation. |
|  | An interprofessional education pilot program in maternity care: findings from an exploratory case study of undergraduate students (Meffe et al., 2012) | Explore how participation in an IPE program in maternity care enhances student knowledge, skills/attitudes, and promotes collaborative behavior in practice | Qualitative | Canada | 25 nursing, midwifery and medical students in the third or final year of their respective programs | Interviews | 6-module IPE program in maternity care for undergraduate nursing, midwifery and medical students and 2 clinical shadowing experiences. The teaching methods used included didactic presentations, readings, videos, discussions, role-plays and debriefing. The program was designed to promote self-directed, student-centred learning, with opportunities for collaborative group work. Teamwork was modelled by the facilitators, who included both IPE experts and maternity care providers from different professions. | The IPE program in maternity care was effective in cultivating collaborative behaviours among undergraduate nursing, midwifery and medical students. The program helped students develop skills in relationship-building, confident communication and willingness to collaborate. The program’s focus on providing woman/family-centred care helped students overcome professional differences and work together. |
|  | Embedding interprofessional education in clinical settings: medical and dental student perceptions of a patient interview-storytelling experience (Miller et al., 2024) | To facilitate student-patient conversations to elicit patient reflections on the health care professionals who keep them healthy and care for them when they are unwell. | Qualitative | USA | 158 medical and dental students | Students’ reflections on patient stories | The key intervention was a 5–10-minute semi-structured patient interview-storytelling experience, where first-year medical and dental students used an interview card with guiding questions to elicit patients’ reflections on the healthcare professionals involved in their care. This was followed by a 1-hour class session where students discussed and reflected on the patient stories and their learnings about interprofessional collaboration. | Students valued patient-centred holistic care as the goal of interprofessional collaboration. Students reflected emerging professional and interprofessional identities in relating to patients, teams and systems. Students appreciated how interprofessional care is complex and challenging, requiring sustained effort and commitment. |
|  | Impact of an interprofessional training ward on interprofessional competencies-a quantitative longitudinal study (Mink et al., 2021) | To assess the development of interprofessional competencies on an interprofessional training ward in terms of communication, cooperation, coordination, teamwork and interprofessional learning. | Quantitative | Germany | 58 medical and 63 nursing students; 56 male and 65 female, age 19 to 40 years | Questionnaires with University of the West of England Interprofessional Questionnaire (UWE-IP) validated German version, Interprofessional Socialization and Valuing Scale (ISVS) and Interprofessional Team Collaboration Scale (AITCS) | A 3-5 week placement of undergraduate medical and nursing students on an interprofessional training ward (IPTW). Students worked in interprofessional tandems of one medical and one nursing student to provide patient care tasks, participate in collaborative activities like ward rounds and handovers and engage in problem-based learning and feedback sessions facilitated by registered nurses and physicians. | The study found statistically significant positive short-term effects in interprofessional competencies in all three questionnaires. There were statistically significant positive long-term effects in the ISVS and the AITCS concerning socialisation and collaboration. There were negative impressions of participants towards interprofessional interaction in clinical practice. |
|  | Medical and pharmacy students shadowing advanced practice nurses to develop interprofessional competencies (Monahan et al., 2018) | To increase medical and pharmacy students’ knowledge of the role of advanced practice nurses (APNs) as members of the interprofessional team and increase medical and pharmacy students’ knowledge of interprofessional collaborative team practices | Quantitative | USA | 20 medical and 20 pharmacy students | Surveys | 4-hour job-shadowing experience where second-year medical and third-year pharmacy students were paired together to shadow an APN at one of three outpatient clinics, a trauma service, cardiology or obstetrics/gynaecology. | The job-shadowing project increased medical and pharmacy students’ knowledge of interprofessional collaborative team practices, perception of the quality of communication between APNs, physicians, and pharmacists and knowledge of the role of APNs as members of the interprofessional team. |
|  | Improving outcomes in adults with diabetes through an interprofessional collaborative practice program (Nagelkerk et al., 2018) | To determine if implementation of an interprofessional collaborative practice (IPCP) program in a Federally Qualified Health Center (FQHC) family practice clinic improves diabetic patient health outcomes, determine if placing a team of students in an IPCP environment increases access to care by increasing the number of patients that can be seen in the clinic, determine if implementation of an IPCP program increases staff and patient satisfaction. | Mixed methods | USA | 10 medical 5 physician assistants and 7 pharmacy students | Interdisciplinary Education Perception Scale (IEPS), the Entry-level Interprofessional Questionnaire (ELIQ), the Collaborative Practice Assessment Tool (CPAT), the National Center Data Repository Network Users Survey, pre and post module knowledge tests, program evaluation surveys and focus group discussions. | IPCP program involving an online IPCP educational program, orientation on IPCP program, integration of medical, pharmacy, and physician assistant students into the care teams, scheduled interprofessional student assignments, shared workspaces and informal case discussions and collaborative care planning with student teams during daily huddles. | The IPCP program led to significant increases in learner knowledge in team dynamics and implementing healthcare behaviour changes. For high-risk diabetic patients, the IPCP program resulted in statistically significant improvements in hgba1c, triglycerides, lipid ratios and blood glucose. Provider productivity, increased after the implementation of the IPCP program and student interprofessional collaborative care teams. |
|  | Case-based interprofessional learning for undergraduate healthcare professionals in the clinical setting (Nasir et al., 2017) | To enable students from different healthcare professional backgrounds to gain a better understanding of each other’s roles, to improve students’ professional competence and seek better outcomes for patients | Mixed methods | UK | 143 Nursing, 139 pharmacy, 28 Medical, 8 physician associate, 7 physiotherapy, 2 midwifery, 1 occupational therapy and 1 speech and language therapy students | Survey | Case-based interprofessional learning (IPL) sessions delivered weekly to small groups of 4 students from 2 or more healthcare professions on 6 different hospital wards covering various clinical specialties. Each student attended one IPL session during the academic year. | The case-based interprofessional learning sessions were well-received by the students, including those with prior IPL experience, and the majority indicated they would change their future professional practice as a result. |
|  | Designing, implementing and sustaining IPE within an authentic clinical environment: the impact on student learning (Naumann, Mullins, et al., 2021a) | To report on changes in students’ attitudes to IPP in response to involvement in the delivery of IPP for type 2 diabetic patients (T2DM) in an authentic, practice-based setting and to explore the reflections of students in response to participation in the delivery of interprofessional care and participation in three structured IPE activities undertaken whilst attending their work-integrated learning placement. | Mixed methods | Australia | 35 exercise physiology, social work, nutrition and dietetic, and nursing students | SPICE-R2 instrument and students’ reflections | 10-week exercise, nutrition and healthy lifestyle education program for T2DM patients | The IPE program led to significant improvements in students’ perceptions of interprofessional education, including their understanding of roles and responsibilities, teamwork, and patient outcomes, provided an authentic learning experience that was best suited for final year students. and enabled students to better understand their own roles and the roles of other health professionals, which is critical for effective interprofessional collaboration. |
|  | Developing the next generation of healthcare professionals: the impact of an interprofessional education placement model (Naumann, Schumacher, et al., 2021b) | To examine the effect of a structured IPE placement program on students’ perceptions of IPE within an authentic healthcare setting and to explore perceptions of IPE and IPP in the practice environment across four different clinical areas | Quantitative | Australia | 20 nursing, 6 speech pathology, 4 physiotherapy, 4 occupational therapy, 1 dietetics and 1 psychology students | SPICE-R2 instrument | 1. A choice of structured IPE activities:  a. Interviewing a team member from another profession  b. Shadowing a team member from another profession  c. Participating in a team meeting 2. A debriefing workshop for students to reflect on their experience | The IPE placement program led to a significant improvement in students’ overall perceptions of interprofessional education. The largest improvements were seen in students’ understanding of professional roles/responsibilities and their perceptions of the impact of interprofessional practice on patient outcomes. Developing an understanding of one’s own professional role and the roles of other health professions was a key learning outcome of the IPE placement program. |
|  | Interprofessional clinical training improves self-efficacy of health care students (Nørgaard et al., 2013) | To assess the impact of interprofessional clinical training on students’ perception of self-efficacy in interprofessional collaboration | Mixed methods | Denmark | 345 nursing, medical, physiotherapy, occupational therapy, laboratory technology and radiography students | Questionnaire | Interprofessional clinical study unit that provided a two-week clinical training program for students from nursing, medicine, physiotherapy, occupational therapy, laboratory technology and radiography professions. | Interprofessional clinical training improved students’ perceived self-efficacy in interprofessional collaboration compared to traditional clinical training. The effect of the interprofessional training was still present at the end of the students’ clinical training period. The effect was most pronounced for the groups with the lowest self-efficacy at baseline. |
|  | A structured approach to intentional interprofessional experiential education at a non-academic community hospital (Nwaesei et al., 2019) | To develop and implement a multimodal structured approach to intentional interprofessional experiential education at a non-academic community hospital, and to evaluate students’ perceptions of interprofessional clinical education targeting IPEC competencies | Quantitative | USA | 18 student pharmacists and 18 medical students; 14 male and 25 female, 18 years and above | Physician-Pharmacist Interprofessional Clinical Education, Version 2 survey | Daily pre-rounds with one-on-one interactions between student pharmacists and medical students to discuss mutual patients and exchange recommendations, daily teaching rounds involving an interdisciplinary team and once or twice weekly lunch-and-learn sessions focused on reviewing and discussing evidence-based guidelines for common internal medicine diseases. This IPE program was implemented over an 8-month period. | Students’ perceptions of IPEC competencies, including values/ethics, roles/responsibilities, and teams and teamwork, significantly improved after the interprofessional experiential education program. The majority of students found the different components of the structured IPE program to be effective or very effective. Students provided more positive feedback about the interprofessional experiential education program. |
|  | Attitudes of dental and chiropractic students towards a shared learning programme-an interprofessional learning model (Omar et al., 2021) | To describes the development and implementation of an IPL model based on ergonomics and work-related musculoskeletal disorders (wmsds) to assess the dental and chiropractic students’ attitude towards the shared IPL model through a pre/postscale. | Quantitative | Malaysia | 46 dental and 23 chiropractic students | Interprofessional learning scale (RIPLS) | An IPE program between dental students and chiropractic students. The program involved didactic lectures for both groups of students, as well as opportunities for them to observe each other’s clinical practices through scheduled visits. | Dental and chiropractic students showed similar levels of readiness for shared learning. The IPL programme contributed to the development of the students’ positive perceptions towards the positive professional identity and the roles of other healthcare professionals. |
|  | A pilot implementation of interprofessional education in a community-academe partnership in the Philippines (Opina-Tan, 2013) | To describe the activities of the interprofessional teams involved in the pilot implementation of the Family Case Management (FCM) initiative, and to present the perceptions of the student participants regarding the most useful and least useful aspects of the FCM experience, as well as their recommendations for improvement. | Mixed methods | Philippines | 5 medical, 8 physical therapy, 2 occupational therapy, 2 speech pathology and 2 nursing students | Questionnaire | Patient and family involvement and consent for the intervention, comprehensive assessment of the patient and family, including medical, functional, social and environmental factors, collaborative development of an intervention plan by the interprofessional team, with input from the patient and family, implementation of the intervention plan, which included medical, educational, and psychosocial components, with training of team members to ensure continuity of care and regular monitoring and adjustment of the intervention plan based on patient and family progress | The student participants generally had a positive experience with the IPE initiative. The most useful aspects were related to learning about collaboration, appreciating each other’s roles, providing holistic care, serving the community and the unique nature of the learning experience. Challenges and limitations included difficulties with coordination and communication, issues with patient management, problems with the program structure and limitations of the community setting. |
|  | Promoting older adult health with interprofessional education through community-based health screening (Ostertag et al., 2022) | To provide clinical training in geriatrics for health professions students through community-based health screenings for older adults improve the overall health of older adults in Montana by providing health information and education | Mixed methods | USA | 138 pharmacy, nursing, physical therapy, medical and social work | Survey and verbal reviews | Participating as interprofessional teams in IPHARM (improving Health Among Rural Montanans), a university-based community health screening program, to address the health of older adults. | Surveys from students indicated that the experience promoted effective interprofessional team skills related to communication, an increased understanding of the roles and responsibilities of the health care team and how to positively impact the health of older adults. |
|  | Interprofessional student hotspotting: preparing future health professionals to deliver team-based care for complex patients (Powers et al., 2022) | To examine how health professions students described their experiences in an interprofessional student hotspotting program and assess the impact of the hotspotting program on students’ perceived ability to provide team-based care to medically and socially complex patients | Qualitative | USA | 7 medical, 7 social work, 5 pharmacy, 4 nursing and 1 health psychology students | Focus group discussions | Interprofessional student hotspotting program that involved teams of 5-7 students from medicine, social work, pharmacy, nursing and health psychology working with 2 assigned patients over a 6-month period to identify needs and design interventions to improve health and reduce healthcare utilisation. The teams met with patients in various settings and conducted regular phone check-ins and attended biweekly 75-minute full-cohort meetings with presentations and teamwork time. | Students observed benefits of interprofessional collaboration in providing care for complex patients, gained skills for collaborative care, such as understanding each other’s roles and responsibilities and improving communication strategies and learnt approaches to caring for complex patients, including developing empathy, addressing social determinants of health, and navigating the fragmented healthcare system. Students experienced challenges in managing group dynamics, such as unequal participation and perceived hierarchies. |
|  | Change in attitudes and perceptions of undergraduate health profession students towards inter-professional education following an educational experience in post-natal care (Ray et al., 2021) | To study the change in students’ perception of comfort level with questioning and being questioned by students of other health care professions and the reliability i.e. Extent to which they can rely on the accuracy of information given by other healthcare professionals after an inter-profession learning experience | Mixed methods | India | 32 medical, 32 nursing and 32 physiotherapy | Questionnaire | Two didactic sessions explaining the concepts and advantages of inter-professional healthcare teams, three sessions explaining the physiological changes during puerperium and the management of postnatal cases, defining the roles of obstetricians, nurses and physiotherapists and small group learning sessions with 6 students (2 from each profession) discussing case vignettes and developing holistic care plans, defining profession-specific roles and responsibilities. | There was a significant improvement in the comfort levels of students in questioning and being questioned by other healthcare professionals after the IPE experience. The extent to which students relied on information from other healthcare professions also increased significantly following the IPE experience. Physiotherapy students showed the greatest improvement in comfort levels, while nursing students demonstrated the maximum increase in reliability on other professionals. |
|  | An interprofessional discharge planning curriculum in the clinical learning environment (Robertson et al., 2022) | To develop an interprofessional learning experience around care transitions in the acute care clinical environment, evaluate the impact of the experience on IPEC competencies, perceptions of teamwork, and professional role formation among nursing and medical students, and assess facilitators and barriers of interprofessional education in the clinical setting | Mixed methods | USA | 4 nursing and 4 medical students | IPEC and Nebraska Interprofessional Education Attitude Scale surveys | 4-week interprofessional learning experience centred on discharge planning for patients on an inpatient medical-surgical unit. Nursing and medical students were paired into dyads and assigned patients to work on. The students used worksheets to gather information about their patients and their discharge planning needs, met over lunch to discuss their patients and prepare a presentation, and then presented their cases and participated in a facilitated discussion about the discharge process and care transitions, guided by faculty. | The study developed and implemented a successful interprofessional learning experience around care transitions within the acute care clinical environment. Aligning subject knowledge prior to the experience to alleviate inequity was critical in contributing to a perceived positive experience. Students enjoyed the lunchtime meeting as a time to prepare for conference and get to know each other. |
|  | Implementation of an interprofessional medication therapy management experience (Schussel et al., 2019) | To implement an IPE in medication therapy management (MTM) and assess the impact of the experience on students’ attitudes and skills about interprofessional collaboration. | Quantitative | USA | 22 medical, nursing, nutrition and pharmacy | Student survey, patient survey, clinical recommendations and informal evaluation | 3-week interprofessional education experience in MTM for health science students, where the students met once per week and collaborated in small groups to conduct MTM consultations for patients with complex chronic conditions. | The IPE experience improved students’ attitudes and skills related to interprofessional collaboration, and patients reported positive experiences with the care they received from the student teams. The implementation of the IPE program led to the UAMMC expanding its interprofessional approach by hiring nursing students to provide MTM services in addition to the existing team of student pharmacists. |
|  | The development of clinical reasoning and interprofessional behaviours: service-learning at a student-run free clinic (Seif et al., 2014) | To examine the benefits of utilising student run-free medical clinics. As a service-learning experience that not only improves access to patient care but also fosters positive interprofessional attitudes and enhances students’ clinical reasoning skills. | Quantitative | USA | 232 pre-clinical occupational therapy, physical therapy, physician assistant, medical and pharmacy students | Survey instruments (Interdisciplinary Education Perception Scale (IEPS), Readiness for Interprofessional Learning Scale (RIPLS) and Self-Assessment of Clinical Reflection and Reasoning (SACRR)) | An interprofessional service-learning course called “Caring for the Community - A Service-Learning Elective” and volunteering at the CARES student-run free clinic, where students were required to participate as student clinicians at least 4 times during the semester-long course. | Students who completed an interprofessional service-learning course and volunteered at a student-run free clinic showed significant improvements in their perceptions of interprofessional behaviours and clinical reasoning skills compared to students who did not participate. No significant differences in interprofessional attitudes between the experimental and control groups were found. |
|  | Assessing interprofessional education collaborative competencies in service-learning course (Sevin et al., 2016) | To investigate the effect of an interprofessional service-learning course on health professions students’ self-assessment of Interprofessional Education Collaborative (IPEC) competencies | Quantitative | USA | 7 nursing, 6 pharmacy and 2 social work students; 4 male and 11 female, 18 years and above | Questionnaire | A service component where students provided patient care at a free clinic and seven 2-hour workshops every other week where students reflected on their experiences and discussed topics like roles, team dynamics, communication and caring for underserved populations. | Students’ self-assessment of interprofessional competencies significantly improved in all four IPEC domains after completing the service-learning course. The largest improvements were seen in the Roles and Responsibilities and Teams and Teamwork domains. |
|  | Evaluation of a student-led interprofessional innovative health promotion model for an underserved population with diabetes: a pilot project (Shiyanbola et al., 2012) | To examine whether students’ knowledge and understanding of diabetes management improved after a student-led interprofessional diabetes health promotion model was implemented and whether students’ awareness of the role of other health professionals in diabetes management improved at the end of the program. | Mixed methods | USA | 47 students from medical, pharmacy, nursing, nutrition and dental hygiene; 26+/-5.38 years | Survey | a 6-month student-led interprofessional health promotion program for patients with diabetes, consisting of 6 monthly 1-2 hour educational sessions covering topics related to diabetes management using the Alphabet Strategy framework. | There were significant improvements in students’ knowledge of diabetes care, understanding of the roles of healthcare professionals and ability to work with other healthcare professionals. There were no significant differences in patients’ diabetes knowledge, understanding of diabetes care and clinical outcomes. |
|  | Determining the impact of an interprofessional learning in practice model on learners and patients (Shrader et al., 2023) | To determine the impact of the interprofessional learning in practice (ILIP) model on student outcomes, specifically student satisfaction and perceived interprofessional collaboration skills and to determine the impact of the ILIP model on patient outcomes. | Quantitative | USA | 87 medical, 15 nursing, 50 pharmacy, 8 dietetics, 14 occupational therapy, 4 physical therapy and 1 psychology students | Survey | The Interprofessional Learning in Practice (ILIP) model, which included an interprofessional practice experience in an ambulatory care setting and an IPE curriculum for students. | Students who experienced the full ILIP model (interprofessional practice experience and intentional IPE curriculum) valued the experience, valued learning from interprofessional preceptors, and gained interprofessional collaboration skills to use in their future practice more than students who only participated in the interprofessional practice experience. The ILIP model was associated with improved patient outcomes related to depression screening and glycaemic control of diabetes. |
|  | Interdisciplinary approach to teaching medication adherence to pharmacy and osteopathic medical students (Singla et al., 2004) | To demonstrate the value of interdisciplinary education to pharmacy and medical students through a medication adherence project | Quantitative | USA | 92 pharmacy and 115 osteopathic medical students | Survey and tablet count | Counselling and distribution of medication distribution | Most pharmacy and medical students had positive attitudes towards interdisciplinary education and working in healthcare teams after participating in the project. Medical students who received more pharmacy counselling and involvement were more satisfied with the interaction and information they received about their medications. Overall medication adherence was 35%, with the group receiving pharmacy counselling and a postcard reminder having the highest adherence rate at 43%. |
|  | A 3-year qualitative evaluation of interprofessional team-based clinical education at an Australian dental school (Storrs et al., 2023) | To explore the contextual meaning related to the previously published quantitative aspects of team-based treatment planning (TBTP) that contributed positively toward oral health students’ interprofessional clinical learning | Qualitative | Australia | 194 dentistry, dental prosthetics, oral health therapy and dental technology students | Online survey and focus group discussions | TBTP program involving students from the dentistry, dental prosthetics, oral health therapy and dental technology programs. Students were organised into clinical teams to collaboratively plan and manage patient care, engaging in experiential learning in clinical and laboratory settings. | Students gained a better understanding of profession-specific and overlapping roles through interprofessional collaboration. The team-based treatment planning process improved students’ communication confidence. Students recognised the team-based treatment planning process as a platform to problem-solve treatment options and advance their understanding of clinical practice, improving their ability to develop treatment plans and learn when to seek assistance from different oral health professionals. |
|  | The effectiveness of community-based interprofessional education for undergraduate medical and health promotion students (Suwanchatchai et al., 2024) | To assess the influence of Community-based interprofessional education (CBIPE) learning on the collaborative competencies of medical and health promotion students. | Mixed methods | Thailand | 193 medical and health promotion students | Interprofessional Collaborative Competencies Attainment Survey (ICCAS Thai version) and semi-structured questionnaire | CBIPE program for undergraduate medical and health promotion students that involved 15 hours of classroom-based theory and 30 hours of fieldwork practice in a rural community over one week. | The CBIPE program significantly improved the collaborative competencies of students. Gender-stratified analysis revealed that women showed improvement in all subscales of the IPE competence scale, while men showed significant improvement in communication, collaboration, conflict management and functioning team skills. The students were highly satisfied with the CBIPE program. |
|  | Effectiveness of an interprofessional education model to influence students’ perceptions on interdisciplinary work (Swinnen et al., 2021) | To evaluate the effect of an IPE session on undergraduate students’ attitudes toward interprofessional collaboration. | Quantitative | Belgium | 200 medical, nursing, physiotherapy and nutrition and dietetics students | Interdisciplinary Education Perception Scale (IEPS) | IPE session where undergraduate students in medicine, nursing, physiotherapy, and nutrition and dietetics worked together in interprofessional groups to prepare a medical history and treatment plan for a patient with breast cancer. The IPE session lasted for a full day, with students first receiving a 30-minute seminar on the patient’s condition, then having at least 1 hour for discussion and preparation and finally presenting their work at the end of the day. | The IPE session led to a statistically significant improvement in students’ perception of their own professional competence and their perception of actual cooperation between different healthcare professions, compared to the control group. |
|  | Different roles same goal: students learn about interprofessional practice in clinical setting (Takahashi et al., 2010) | To formally integrate IPE into the clinical setting in spinal bifida clinic. | Qualitative | Canada | Nursing, physiotherapy and occupational therapy | Questionnaires | IPE program introductory session and participation in spina bifida clinical days. | Students reported increased understanding of their own and others’ roles and a more holistic view of patients and families and demonstrated their ability to work in teams to create collaborative care plans. Clinic team members enjoyed participating in the program, were impressed by the students’ collaborative work, and became more aware of interprofessional issues in their own practice. |
|  | Designing and evaluating an interprofessional practice experience involving dental and pharmacy students (Theodorou et al., 2018) | To prepare pharmacy and dental students to collaborate as members of an interprofessional team by participating in an interprofessional practice experience, to evaluate pharmacy student performance in interprofessional collaboration using a standardised assessment rubric and to evaluate pharmacy and dental student perceptions of the interprofessional practice experience. | Quantitative | USA | 37 pharmacy and 92 dental students | Student Perceptions of Interprofessional Clinical Education-Revised (SPICE-R) Instrument | Pharmacy and dental students collaboratively conducting medical histories and providing tobacco cessation education within a dental admissions clinic. Pharmacy students taking the lead on completing medication histories, performing medication reconciliation and resolving medication discrepancies, while dental students observed and contributed their own thoughts. Dental students completing other medical history components, oral examinations and demonstrating oral cancer screenings, while pharmacy students observed. On interprofessional care days, dental students referring tobacco users to pharmacy students who then provided tobacco cessation education using the 5-As method. | Pharmacy students met or exceeded performance expectations in the interprofessional practice experience. There was a statistically significant increase in student perceptions of interprofessional collaboration for both pharmacy and dental students after the experience. Most pharmacy and dental students and faculty agreed that the learning objectives of the practice experience were achieved. |
|  | Evaluation of interprofessional training in home care (Törnkvist & Hegefjärd, 2008) | To evaluate undergraduate students’ experiences of a one-day interprofessional home care training in a realistic setting compared to students who had uniprofessional placements and to investigate whether the interprofessional home care training was a successful way to help students develop an understanding of teamwork. | Mixed methods | Sweden | 7 dietician, 26 district nursing, 90 nursing, 45 occupational therapy and 183 physiotherapy students; 47 male and 304 female | Questionnaire | One-day interprofessional home care training program for a group of undergraduate students from multiple healthcare professions. The training took place in a realistic home care setting and involved working through a fictitious patient case in a structured format including professional group work, a team conference, interprofessional group work and a final reflection session. | The students who participated in the one-day interprofessional home care training reported higher levels of understanding and satisfaction compared to the control group on various aspects of interprofessional teamwork and patient care. |
|  | Are the stars aligned? Healthcare students’ conditions for negotiating tasks and competencies during interprofessional clinical placement. (Törnqvist et al, 2023) | To explore students’ negotiation of tasks and competencies when students are working together as an interprofessional team during clinical placement. | Qualitative | Sweden and Norway | Final-year healthcare students | Fieldnotes, informal conversations, group, and focus group interviews. | Healthcare students worked together with between one to three patients during their IPE clinical placement. The training took place at two different sites: a health centre (HC) and an interprofessional training ward (IPTW) | The study showed that final-year healthcare students can negotiate who in the team has the competence suited for a specific task. The findings imply that educators need to be attentive to this and make an effort to ensure that students benefit from these intersecting communities of practice, both when they align and when they are in conflict. |
|  | Interprofessional training enhances collaboration between nursing and medical students: a pilot study (Turrentine et al., 2016) | To enhance interprofessional education between nursing and medical students, provide an opportunity for students to practice interprofessional geriatric assessment skills, move the learning experience from simulation to a real clinical environment and better prepare students to integrate into interdisciplinary clinic teams | Mixed methods | USA | 17 medical and nursing students | Surveys | An initial 1-2 hour education session where students observed a video, 2-hour practice sessions where students worked in nurse-physician pairs to complete the geriatric assessment on standardised patients and a final clinic session where students conducted the geriatric assessment independently or in nurse-physician pairs on real patients. | There was a significant improvement in students’ geriatric assessment knowledge from pre-test to post-test scores. Students who performed the final clinic assessment in nurse/physician pairs had significantly higher evaluation scores compared to those who performed the assessment individually. |
|  | Analysis of an interprofessional home visit assignment: student perceptions of team-based care, home visits, and medication-related problems (Vaughn et al., 2014) | To evaluate changes in student attitudes and self-assessed skills and abilities related to interprofessional team-based patient care, home visits and identification of medication-related problems | Mixed methods | USA | 22 medical and 20 pharmacy students | Survey | An interprofessional home visit assignment for medical students and pharmacy students. The students worked in pairs to identify a patient at risk for medication-related problems, schedule a home visit, and conduct a medication-focused interview with the patient while accompanied by a clinical pharmacy preceptor. After the visit, the student teams discussed potential medication-related problems with a clinical pharmacist preceptor, wrote a SOAP note for the patient’s physician and completed an individual reflection paper. | Students’ self-perception of skills and abilities related to interprofessional team-based care and identification of medication-related problems improved after completing the IPE medication-focused home visit assignment. The students showed significant improvements across 12 survey items related to their self-rated skills and abilities. Student feedback on the IPE home visit experience was overwhelmingly positive, and students frequently stated they would recommend the experience to other students. |
|  | Building great health care teams: enhancing interprofessional work readiness skills, knowledge and values for undergraduate health care students (Venville & Andrews, 2020) | Develop interprofessional work readiness skills, knowledge and values in undergraduate health professional students. Evaluate the impact of the IPE program on participants’ interprofessional skills, knowledge, beliefs, values, attitudes and confidence. Determine the sustainability of the IPE program. | Quantitative | Australia | 24 social work, occupational therapy, physiotherapy, speech pathology, nursing, psychology, dietetics | The Work Self-Efficacy Inventory (WS-Ei) and interprofessional socialisation and valuing scale (ISVS) | Students working in interprofessional teams of 8 members from different disciplines over 3 days, with facilitated team-based learning activities. | Participation in the IPE program resulted in substantial shifts in knowledge, skills and values for the undergraduate health care students. The program was effective in teaching the affective aspects of interprofessional practice, such as teamwork and dealing with challenging relational and behavioural issues in teams. The program highlighted the patient voice and provided authentic learning opportunities for students to apply advanced teamwork skills, appropriate and effective interprofessional communication and patient/client-centred practice. |
|  | Students’ motivation for interprofessional collaboration after their experience on an IPE ward: a qualitative analysis framed by self-determination theory (Visser et al., 2019) | To investigate which elements of IPE in a clinical ward potentially influence students’ feelings in autonomy (feeling of choice), competence (feeling of capability) and relatedness (feeling of belonging) | Qualitative | Netherlands | 10 medical, 5 nursing, 2 pharmacy and 4 physical therapy; 5 male and 16 female | Interviews | A team of students from different healthcare professions responsible for the care of preselected patients, under close supervision. | The IPE ward experience enhanced students’ autonomous motivation for interprofessional collaboration by fulfilling their needs for autonomy, competence and relatedness. Students valued the opportunity to learn about the roles, responsibilities and clinical reasoning of other professions. |
|  | Students’ experiences and perceptions of interprofessional education during rural placement: a mixed methods study (Walker et al., 2019) | To report the student perspective, experience and perception of IPE, a subset of a larger Australian multi-perspectival study conducted to examine the opportunities and application of IPE in rural clinical learning environments. | Mixed methods | Australia | 60 dentistry, occupational health, physiotherapy and occupational therapy; medical, nursing and midwifery; 10 male and 50 female | Survey (Readiness for Interprofessional Learning Scale, Interdisciplinary Education Perception Scale and focused interprofessional questions), interviews and focus group discussions | Rural clinical placement | Students reported numerous opportunities for IPE during rural clinical placements, and most had positive attitudes towards IPE. Some students expressed uncertainty about their professional roles and the need for cooperation with other professions. |
|  | Use of profession-role exchange in an interprofessional student team-based community health service-learning experience (Wang et al., 2020) | To develop a comprehensive and multi-dimension extracurricular IPE model through designing and integrating a profession-role exchange component, that was medical students as pharmacists or nurses, pharmacy students as physicians or nurses, and nursing students as physicians or pharmacists in the interprofessional health-care student team, into the service learning experience in a real community setting. | Quantitative | China | 20 medical, 20 pharmacy and 20 nursing students | Questionnaires with Physician-pharmacist collaboration survey instrument, Physician-nurse collaboration survey instrument, Nurse-pharmacist collaboration survey instrument and “Roles and responsibilities” subscale of Readiness for Interprofessional Learning Scale (RIPLS) | The IPE activity was the “profession-role exchange” component, where students from different healthcare professions were required to play the roles and responsibilities of students from other professions during the community-based interprofessional healthcare service-learning experience. | The addition of a profession-role exchange component resulted in a significant increase in students’ positive attitudes towards interprofessional collaboration compared to the control group. The intervention enhanced students’ role awareness and understanding of the roles of other healthcare professionals and allowed students to experience the perspectives of their partners in the clinical teamwork. |
|  | Placement development teams and interprofessional education with healthcare students (Williamson et al., 2011) | To investigate the impact of a new structure for supporting students and mentors in practice placements (Placement Development Teams) in fostering interprofessional education from the perspective of non-medical health care students and staff. | Qualitative | UK | 41 physiotherapy, adult nursing, podiatry, occupational therapy and paramedicine students | Interviews and focus group discussions | Introduction of Placement Development Teams to support students and mentors from multiple healthcare professions in practice placement areas. | Students had mixed views on the value of interprofessional education support, with some seeing limited benefit from staff of other professions providing informational support. Staff fostered IPE through effective communication and facilitation across different professional groups. |
|  | Community-based interprofessional education in rural primary care (Wros et al., 2023) | To accelerate students’ acquisition of teamwork competencies, specifically in team effectiveness, team communication, reflective practice and situational awareness and to immerse students in community-based rural primary care practice with patients who have high rates of chronic illness and adverse social determinants of health. | Mixed methods | USA | 38 students: 12 physician assistant, 11 medical, 8 family nurse practitioner, and 7 pharmacy; 76% female; 87% under 29 years of age | Assessment for Collaborative Environments (ACE-15) | The IPE activity was the Reaching Rural Residents with Interprofessional Education (R3 IPE) program, which involved health professions students (from family nurse practitioner, medicine, physician assistant, and pharmacy programs) participating in a 4-week rural clinical rotation. As part of this rotation, students spent 6-8 hours per week collaborating with a Project Coordinator and Interprofessional Preceptor to identify and conduct home visits with older adult patients who had complex medical and social needs. The home visits were conducted by teams of 2-4 students. | The ACE-15 teamwork measure showed a significant improvement in students’ teamwork competencies from the beginning to the end of the 4-week rotation. Preceptor observations indicated that students demonstrated strong leadership abilities, professional demeanour and good rapport with patients, though some occasional nervousness and insensitivity to team members were also observed. Student self-reflections were largely positive, with themes of appreciation for working in a collaborative team, learning from other professions, recognising the value of a team approach for patient care and developing compassion for patients. |
|  | Call the on-call: authentic team training on an interprofessional training ward -a case study (Zelić et al., 2023) | To present a learning activity that prepares healthcare students for authentic teamwork where team members are separated into different locations and explore how this setting of separated team members affects student learning, including professional and interprofessional learning as well as team development | Mixed methods | Sweden | 109 nursing, 25 physiotherapy, 50 medical and 14 occupational therapy students | Surveys, notes from reflection sessions, a focus group interview and field observations. | Students from four professions were taking care of patients by working side by side in two teams for two weeks taking care of patients who underwent planned orthopaedic surgeries. The study involved splitting up the student healthcare team, with medical students following the on-call orthopaedic surgeon and the other students remaining on the ward and practising interprofessional telephone communication with nursing students calling the medical students to consult them, using simulated patient cases. | The learning activity did not impair interprofessional learning or team building, and instead created new opportunities for interprofessional learning and team development. Students across all professions viewed the authenticity and relevance of the learning activity as key strengths, which motivated them to engage with the challenges. The study found that splitting up the student team did not weaken the healthcare team. |
