# Supplementary material for: A scoping review of interprofessional education in healthcare: evaluating competency development, educational outcomes and challenges
Source: BMC Med Educ. 2025 Mar 20;25:409. doi: 10.1186/s12909-025-06969-3 (PMC11924666; doi:10.1186/s12909-025-06969-3)
Supplement: Supplementary file 2 — Supplementary Material 2. [file 12909_2025_6969_MOESM2_ESM.docx]

*Supplementary Table 2: Core Competencies developed and educational outcomes*

| **No** | **Title** | **Core Competencies Developed and Positive Outcomes** | **Negative Outcomes/Challenges** |
| --- | --- | --- | --- |
|  | Interprofessional primary healthcare student placements: qualitative findings from a mixed-method evaluation (Aggar et al., 2020) | Broadened scope of practice, Improved teamwork skills | Not reported |
|  | Hotspotting in home health: the impact of interprofessional student-team home visits on readmission rates of super-utilizers of the health care system (Alderman, 2022) | Improved health outcome due to teamwork | Not reported |
|  | Learning together in practice: an interprofessional education programme to appreciate teamwork (Anderson & Thorpe, 2010) | Patient-centred approach to care co-ordination, Clarity of roles and responsibilities, Appreciation of complexity of teamwork | Not reported |
|  | The longitudinal elderly person shadowing program: outcomes from an interprofessional senior partner mentoring program (Basran et al., 2012) | Patient centred care with improved communication with seniors | Not reported |
|  | Increasing reach of the diabetes prevention program in African American churches: project fit lessons learned in using an interprofessional student service-learning approach (Berkley-Patton et al., 2021) | Understanding and appreciation for other health professionals | Conflicting schedules |
|  | The interprofessional care access network (I-CAN): achieving client health outcomes by addressing social determinants in the community (Bradley et al., 2023) | Improvements in health-related outcomes for clients | Not reported |
|  | Fostering interprofessional geriatric patient care skills for health professions students through a nursing facility-based immersion rotation (Byerly et al., 2020) | Identify the roles of interprofessional team members at a nursing home, create team-driven patient-centred care plans, work cooperatively as a part of an interprofessional team, express my knowledge effectively within an interprofessional team, express my opinions effectively within an interprofessional team. | Need for less time in training and having equal participation from students across their interprofessional teams. |
|  | Teaching teamwork: an evaluation of an interprofessional training ward placement for health care students (Cant et al., 2014) | Understanding of other professional roles, communication and shared knowledge, interprofessional teamwork/collaboration, autonomy | Not reported |
|  | Development and evaluation of an interprofessional seminar pilot course to enhance collaboration between health professions at a student-run clinic for underserved populations (Caratelli et al., 2020) | Interprofessional Communication (Promote effective communication among members of an interprofessional (IP) team, Use an IP team approach with the patient to assess the health situation), Values and Ethics (Take into account the ideas of IP team members), Roles and Responsibilities (Understand the abilities and contributions of IP team members), Team and Teamwork (Develop an effective care plan with IP team members) | Not reported |
|  | Evaluating the health “hubs and spokes” interprofessional placements in rural New South Wales, Australia (Craig et al., 2014) | Own competence and autonomy, perception of actual cooperation, understanding others’ value, community/rural focus, working in teams, professional perspectives and roles | Perceived need for cooperation decreased |
|  | The positive impact of interprofessional education: a controlled trial to evaluate a programme for health professional students (Darlow et al., 2015) | Attitudes towards interprofessional teams and learning, students' self-reported effectiveness as interprofessional team members, students' confidence, knowledge and ability to manage long-term condition | Not reported |
|  | Longitudinal impact of preregistration interprofessional education on the attitudes and skills of health professionals during their early careers: a nonrandomised trial with 4-year outcomes (Darlow et al., 2022) | Learning and support received from other health practitioners, better patient care, experiences and outcomes | Not reported |
|  | Local to global: working together to meet the needs of vulnerable communities (Dressel et al., 2017) | Increase in students understanding of how to work together, Increased proficiency in integrating knowledge and experience from other team members, Active listening skill | Not reported |
|  | Interprofessional clinical training for undergraduate students in an emergency department setting (Ericson et al., 2012) | Own role clarity, knowledge and understanding of the other two professions, | Not reported |
|  | Initiation of a multidisciplinary summer studentship in palliative and supportive care in oncology (Fairchild et al., 2012) | Positive professional identity, understand other professions, collaboration, respect, teamwork, positive about contributions | Not reported |
|  | One site fits all? A student ward as a learning practice for interprofessional development (Falk et al., 2013) | Roles and responsibilities- For the nursing students, the descriptions of the enactments of organization and administrative planning of the daily work at the ward stood out as an important and ‘‘expected’’ professional responsibility | The practice architectures of the IPTW, such as the organisation of ward rounds and the requirement for all students to be present, can enable or constrain students’ enactment of their expected professional responsibilities |
|  | Mixed methods study: a one-week international service project enhances healthcare competencies (Fell et al., 2019) | Respect the unique cultures, values, roles/responsibilities, and expertise of other health professions. Communicate with team members to clarify each member’s responsibility in executing components of a treatment plan or  public health intervention | Not reported |
|  | Interprofessional education for complex neurological cases (Fenn et al., 2020) | Higher skills in operating within interprofessional teams, valuing interprofessional teamwork, stronger beliefs in the efficiency of interprofessional teams | Not reported |
|  | Experiences from an interprofessional student-assisted chronic disease clinic (Frakes et al., 2014) | Discipline-specific knowledge, understanding of interprofessional practice and communication skills | Not reported |
|  | SIESTA: a quick interprofessional learning activity fostering collaboration and communication between paediatric nursing trainees and medical students (Friedrich et al., 2021) | Communication, teamwork, knowledge of professional roles | Not reported |
|  | An interprofessional health assessment program in rural amateur sport (Grace & Coutts, 2017) | Teamwork and collaboration, professional identity, Positive Professional Identity, Roles and Responsibilities, patient-centred care | Negative Personal Identity |
|  | Effects of interprofessional education on patient perceived quality of care (Hallin et al., 2011) | Higher quality of care, participation in treatment decisions, satisfaction with information about home assistance and feeling that patient's home situation was taken into account | Not reported |
|  | A safe place with space for learning: experiences from an interprofessional training ward (Hallin & Kiessling, 2016) | Collaboration, role clarity, teamwork | If the learning environment is impaired, students’ development may be limited to only personal and/or professional development, lacking interprofessional competence and a comprehensive view of practice. |
|  | Active interprofessional education in a patient-based setting increases perceived collaborative and professional competence (Hallin et al., 2009) | Role clarity, patient-centred care, communication and teamwork | Not reported |
|  | Following the growth of Sarah’s baby: an interprofessional education activity for medical nutrition education and diagnostic medical sonography students (Hanson et al., 2017) | Working with students from another health professions, own role clarity, understanding of other profession's roles, improved health outcome due to teamwork | Not reported |
|  | Students’ perceptions of interprofessional education in geriatrics: a qualitative analysis (Holmes et al., 2020) | Interprofessional collaboration, group dynamics, role clarity | Not reported |
|  | Evaluation of interprofessional student teams in the emergency department: opportunities and challenges (Hood et al., 2022) | Understanding of the role and function of other disciplines in health care, felt comfortable in asking for advice or assistance when necessary from student colleagues, comfortable taking the lead in a student group, comfortable sharing responsibility for delivery of health care, comfortable putting forward my opinions in a group, better understanding of the patient’s role in healthcare decision-making, comfortable communicating with patients and their families to seek their input into care, working with other students from different professions to: form a team, resolve problems in the team, develop a realistic appropriate patient care plan, understand our respective roles in an interprofessional team, understand the benefits to patients of team care. | Challenges were identified in the organisation and supervision of the student teams, with a lack of consistency in approaches to supervision among professional staff. |
|  | Outcomes from a single-intervention trial to improve interprofessional practice behaviours at a student-led free clinic (Horbal et al., 2019) | All three composites, “ability to work with others,” “value in working with others,” and “comfort in working with others” showed noteworthy differences in control versus intervention days. | Not reported |
|  | “I learned that I am loved”: older adults and undergraduate students mutually benefit from an interprofessional service-learning health promotion program (Howell et al., 2021) | Respect and shared values, understand my own and other professions roles and limitations, communicate more effectively with patients/clients  and other professionals, teamwork, patient centred care and Collaboration | Not reported |
|  | Students’ approaches to learning in clinical interprofessional context (Hylin et al., 2011) | Understanding of their own profession, knowledge about each other’s professions | Not reported |
|  | Interprofessional training in clinical practice on a training ward for healthcare students: a two-year follow-up (Hylin et al., 2007) | Development of independence and self-responsibility, strengthened understanding of professional roles, Increased self-esteem, Improved teamwork | Difficulties in developing professional roles and professional identity, and too few profession-specific tasks for medical students |
|  | A rural interprofessional educational initiative: what success looks like (Jackman et al., 2016) | enhanced knowledge of each other’s scope of practice, firsthand insights into rural interprofessional teamwork, and increased confidence in working with professionals from other disciplines. | Barriers to success included logistical challenges, resistance from some staff, and student discomfort with interprofessional interactions. |
|  | Spreading the concept: an attempt to translate an interprofessional clinical placement across a Danish hospital (Jakobsen & Hansen, 2014) | Collaboration, understanding of other profession's roles, equality, communication, professional identity | Not reported |
|  | The interprofessional learning experience: findings from a qualitative study based in an outpatient setting (Jakobsen et al., 2017) | Professional identity | Not reported |
|  | Emotions and clinical learning in an interprofessional outpatient clinic: a focused ethnographic study (Jakobsen et al., 2019) | Equality, communication, and role distribution | Negative emotions due to unexpected incidences |
|  | Pharmacy and medical student interprofessional education placement week (Jebara et al., 2022) | Students learned more about patient care in general and about each other's profession. Students were better prepared for a more collaborative practice in future. Placement improved student's learning experience | Not reported |
|  | Interprofessional education in practice (Joseph et al., 2012) | Clarity of roles and responsibilities, Teams and Teamwork | Not reported |
|  | Students’ perceptions of interprofessional collaboration on the care of diabetes: a qualitative study (Kangas et al., 2021) | Understanding the patient’s point of view better, team collaboration, understanding own and others' roles, respect, communication | Not reported |
|  | An interprofessional patient assessment involving medical and nursing students: a qualitative study (Kara et al., 2018) | Teamwork, collaboration, patient-centred care | Apprehension and of feeling intimidated or nervous initially |
|  | The mixed-discipline aged-care student clinic: an authentic interprofessional learning initiative (Kent et al., 2014) | Patient-centred care, role clarification, teamwork, verbal and written communication skills | Not reported |
|  | Interprofessional education in a community-based setting: an opportunity for interprofessional learning and collaboration (Keshmiri & Barghi, 2021) | Interprofessional learning, teamwork and collaboration | Not reported |
|  | The lived experience of health sciences students’ participation in an interprofessional community-based stroke class (Kloppers et al., 2022) | Own and others' scope of practice, teamwork and collaboration, value of person-centred functional outcomes | Not reported |
|  | Capturing students’ learning experiences and academic emotions at an interprofessional training ward (Lachmann et al., 2013) | Collaboration in the team | Students reported experiences of higher stress levels on the first day of the IPTW course |
|  | Fostering a culture of interprofessional education for radiation therapy and medical dosimetry students (Lavender et al., 2014) | Comfortable communicating with attending physicians, residents, physicists and faculty | Not reported |
|  | Students’ learning experiences from interprofessional collaboration on a training ward in municipal care (Lidskog et al., 2008) | Collaboration, teamwork, own and others' professional roles | Not reported |
|  | What and how do students learn in an interprofessional student-run clinic? An educational framework for team-based care (Lie et al., 2016) | Recognition of other professions’ roles and scope of practice, appreciation of benefits of team-based care, recognition of the role of advocacy-/systems-based care, self-improvement in leadership and clinical skills | Not reported |
|  | Implementation and evaluation of a community-based interprofessional learning activity (Luebbers et al., 2017) | Communication, Collaboration,  Roles and responsibilities, Collaborative patient/family-centred approach, Conflict management/resolution and  Team functioning | Not reported |
|  | Interprofessional clinical training in mental health improves students’ readiness for interprofessional collaboration: a nonrandomized intervention study (Marcussen et al., 2019) | Teamwork and Collaboration" and "Positive Professional Identity, Shared decision making, Cooperation and Coordination | Not reported |
|  | Interprofessional training for final year healthcare students: a mixed methods evaluation of the impact on ward staff and students of a two-week placement and of factors affecting sustainability (McGettigan & McKendree, 2015) | Increased understanding of other profession, Teamwork and collaboration | Not reported |
|  | Australian evidence for interprofessional education contributing to effective teamwork preparation and interest in rural practice (McNair et al., 2005) | Respect, interprofessional collaboration, competency | Not reported |
|  | An interprofessional education pilot program in maternity care: findings from an exploratory case study of undergraduate students (Meffe et al., 2012) | Communication, collaboration, patient-centred care | Not reported |
|  | Embedding interprofessional education in clinical settings: medical and dental student perceptions of a patient interview-storytelling experience (Miller et al., 2024) | Patient-centred holistic care, professional and interprofessional identities | Not reported |
|  | Impact of an interprofessional training ward on interprofessional competencies-a quantitative longitudinal study (Mink et al., 2021) | Communication and Teamwork, Interprofessional Learning, Interprofessional Interaction, Partnership, cooperation, coordination | Not reported |
|  | Medical and pharmacy students shadowing advanced practice nurses to develop interprofessional competencies (Monahan et al., 2018) | Role and responsibility clarity, Collaboration, Communication | Not reported |
|  | Improving outcomes in adults with diabetes through an interprofessional collaborative practice program (Nagelkerk et al., 2018) | Improved communication, Teamwork, Patient Safety | Not reported |
|  | Case-based interprofessional learning for undergraduate healthcare professionals in the clinical setting (Nasir et al., 2017) | Collaboration, roles and responsibilities | Not reported |
|  | Designing, implementing and sustaining IPE within an authentic clinical environment: the impact on student learning (Naumann, Mullins, et al., 2021) | Interprofessional Teamwork and Team-based Practice, Roles/Responsibilities for Collaborative Practice, Patient Outcomes from Collaborative Practice | Not reported |
|  | Developing the next generation of healthcare professionals: the impact of an interprofessional education placement model (Naumann, Schumacher, et al., 2021) | Roles and responsibilities, patient outcomes, teamwork | Not reported |
|  | Interprofessional clinical training improves self-efficacy of health care students (Nørgaard et al., 2013) | Collaboration, identifying other professions’ functions concerning inpatients, communication | Not reported |
|  | A structured approach to intentional interprofessional experiential education at a non-academic community hospital (Nwaesei et al., 2019) | Teamwork, role clarity, patient centred care | Not reported |
|  | Attitudes of dental and chiropractic students towards a shared learning programme-an interprofessional learning model (Omar et al., 2021) | Professional identity, roles and responsibilities | Not reported |
|  | A pilot implementation of interprofessional education in a community-academe partnership in the Philippines (Opina-Tan, 2013) | learning about collaboration, appreciation of roles, holistic care, service to the community | Challenges and limitations included difficulties with coordination and communication, issues with patient management, problems with the program structure and limitations of the community setting. |
|  | Promoting older adult health with interprofessional education through community-based health screening (Ostertag et al., 2022) | Communication, patient viewpoint | Not reported |
|  | Interprofessional student hotspotting: preparing future health professionals to deliver team-based care for complex patients (Powers et al., 2022) | Pulling various perspectives and ideas together for holistic care, formulation of innovative solutions, clarity of roles and responsibilities | Students experienced challenges in managing group dynamics, such as unequal participation and perceived hierarchies. |
|  | Change in attitudes and perceptions of undergraduate health profession students towards inter-professional education following an educational experience in post-natal care (Ray et al., 2021) | Teamwork, communication, knowledge of other professionals | Not reported |
|  | An interprofessional discharge planning curriculum in the clinical learning environment (Robertson et al., 2022) | Teamwork, roles and responsibilities, collaboration | The medical students who participated were required to leave their team and forego patient care to participate in this IPE activity. Inequity issues were identified. |
|  | Implementation of an interprofessional medication therapy management experience (Schussel et al., 2019) | Engage diverse healthcare professionals, use the full scope of knowledge, skills, and abilities of available health professionals and healthcare workers, express my knowledge and opinions to team members involved in patient care with confidence, clarity and respect and apply leadership practices that support collaborative practice and team effectiveness | Not reported |
|  | The development of clinical reasoning and interprofessional behaviours: service-learning at a student-run free clinic (Seif et al., 2014) | Teamwork, conflict resolution | Not reported |
|  | Assessing interprofessional education collaborative competencies in service-learning course (Sevin et al., 2016) | Values and ethics, roles and responsibilities, interprofessional communication, and teams and teamwork | Not reported |
|  | Evaluation of a student-led interprofessional innovative health promotion model for an underserved population with diabetes: a pilot project (Shiyanbola et al., 2012) | Ability to work with other healthcare professionals, ability to work with patient to change their health behaviours | Not reported |
|  | Determining the impact of an interprofessional learning in practice model on learners and patients (Shrader et al., 2023) | Interprofessional collaborative skills | Not reported |
|  | Interdisciplinary approach to teaching medication adherence to pharmacy and osteopathic medical students (Singla et al., 2004) | Improved patient health outcomes | Not reported |
|  | A 3-year qualitative evaluation of interprofessional team-based clinical education at an Australian dental school (Storrs et al., 2023) | Role learning, communication confidence, Pro-active collaborative teamwork | Disrespecting professional roles of some professions by other students in the IPE team, Uncertain communication abilities, Ineffective teams |
|  | The effectiveness of community-based interprofessional education for undergraduate medical and health promotion students (Suwanchatchai et al., 2024) | Communication, collaboration, roles and responsibilities, collaborative family-centred approach, conflict management/resolution, team functioning | Not reported |
|  | Effectiveness of an interprofessional education model to influence students’ perceptions on interdisciplinary work (Swinnen et al., 2021) | Perception of competence in one’s own profession” and “perception of actual cooperation | Not reported |
|  | Different roles, same goal: students learn about interprofessional practice in clinical setting (Takahashi et al., 2010) | Roles and responsibilities, holistic patient care, collaborative care | Extra time requirement |
|  | Designing and evaluating an interprofessional practice experience involving dental and pharmacy students (Theodorou et al., 2018) | Improved values and ethics; roles and responsibilities; communication skills; Teams and teamwork; | Not reported |
|  | Evaluation of interprofessional training in home care (Törnkvist & Hegefjärd, 2008) | Understanding for other’s professional responsibilities for a patient in home care, understanding for interprofessional teamwork in home care | Not reported |
|  | Are the stars aligned? Healthcare students’ conditions for negotiating tasks and competencies during interprofessional clinical placement. (Törnqvist, et al., 2023) | Feelings of being safe and having time to negotiate enabled students to discern their respective competencies and conclude what to do and who should do it | Hindered or interrupted negotiations with unaligned communities of practice |
|  | Interprofessional training enhances collaboration between nursing and medical students: a pilot study (Turrentine et al., 2016) | Increased knowledge of other professions, Identification of decision-making capacity. | Not reported |
|  | Analysis of an interprofessional home visit assignment: student perceptions of team-based care, home visits, and medication-related problems (Vaughn et al., 2014) | Teamwork, role clarity, communication, appreciation of patient perspective | Not reported |
|  | Building great health care teams: enhancing interprofessional work readiness skills, knowledge and values for undergraduate health care students (Venville & Andrews, 2020) | Teamwork, problem solving, role clarity | Not reported |
|  | Students’ motivation for interprofessional collaboration after their experience on an IPE ward: a qualitative analysis framed by self-determination theory (Visser et al., 2019) | Patient-centred care, roles and responsibilities, collaboration, teamwork, communication, feedback | Hierarchy (difference in power) |
|  | Students’ experiences and perceptions of interprofessional education during rural placement: a mixed methods study (Walker et al., 2019) | Collaboration, teamwork | Some students expressed uncertainty about their professional roles and the need for cooperation with other professions |
|  | Use of profession-role exchange in an interprofessional student team-based community health service-learning experience (Wang et al., 2020) | Roles/responsibilities for collaborative practice, role awareness and understanding of the roles of other healthcare professionals, Shared authority, Shared education and teamwork | Not reported |
|  | Placement development teams and interprofessional education with healthcare students (Williamson et al., 2011) | Effective communication and facilitation across different professional groups | Students had mixed views on the value of interprofessional education support, with some seeing limited benefit from staff of other professions providing informational support |
|  | Community-based interprofessional education in rural primary care (Wros et al., 2023) | Teamwork, collaboration, patient-centred care, respect | Occasional nervousness, insensitivity to team members |
|  | Call the on-call: authentic team training on an interprofessional training ward -a case study (Zelić et al., 2023) | Understanding of professional roles during on call hours, communication, teamwork | Not reported |
